# Reversing frailty in older adults: a scoping review

**DOI:** 10.1186/s12877-023-04309-y

**Published:** 2023-11-17

**Authors:** Aurélie Tonjock Kolle, Krystina B. Lewis, Michelle Lalonde, Chantal Backman

**Affiliations:** 1 School of Nursing, Faculty of Health Sciences, University of Ottawa, Ottawa, ON Canada; 2 The Ottawa Hospital Research Institute, Ottawa, ON Canada; 3 University of Ottawa Heart Institute, Ottawa, ON Canada; 4 Institute du Savoir Montfort, Montfort Hospital, Ottawa, ON Canada; 5 Bruyère Research Institute, Ottawa, ON Canada

**Keywords:** Multi component interventions, Single component interventions, Reverse frailty, Frailty domains

## Abstract

**Background:**

Individuals 65 years or older are presumably more susceptible to becoming frail, which increases their risk of multiple adverse health outcomes. Reversing frailty has received recent attention; however, little is understood about what it means and how to achieve it. Thus, the purpose of this scoping review is to synthesize the evidence regarding the impact of frail-related interventions on older adults living with frailty, identify what interventions resulted in frailty reversal and clarify the concept of reverse frailty.

**Methods:**

We followed Arksey and O’Malley’s five-stage scoping review approach and conducted searches in CINAHL, EMBASE, PubMed, and Web of Science. We hand-searched the reference list of included studies and conducted a grey literature search. Two independent reviewers completed the title, abstract screenings, and full-text review using the eligibility criteria, and independently extracted approximately 10% of the studies. We critically appraised studies using Joanna Briggs critical appraisal checklist/tool, and we used a descriptive and narrative method to synthesize and analyze data.

**Results:**

Of 7499 articles, thirty met the criteria and three studies were identified in the references of included studies. Seventeen studies (56.7%) framed frailty as a reversible condition, with 11 studies (36.7%) selecting it as their primary outcome. Reversing frailty varied from either frail to pre-frail, frail to non-frail, and severe to mild frailty. We identified different types of single and multi-component interventions each targeting various domains of frailty. The physical domain was most frequently targeted (n = 32, 97%). Interventions also varied in their frequencies of delivery, intensities, and durations, and targeted participants from different settings, most commonly from community dwellings (n = 23; 69.7%).

**Conclusion:**

Some studies indicated that it is possible to reverse frailty. However, this depended on how the researchers assessed or measured frailty. The current understanding of reverse frailty is a shift from a frail or severely frail state to at least a pre-frail or mildly frail state. To gain further insight into reversing frailty, we recommend a concept analysis. Furthermore, we recommend more primary studies considering the participant’s lived experiences to guide intervention delivery.

**Supplementary Information:**

The online version contains supplementary material available at 10.1186/s12877-023-04309-y.

## Background

Within the next few decades, the population of people aged 65 and over will continue to rise more than all other age groups, with roughly one in six people over 65 by 2050, compared to one in eleven in 2019 [1]. Individuals over 65 years are presumably at greater risk of becoming frail [2–4]. Theoretically, frailty is considered a clinically recognized state of vulnerability that results from an age-related decline in reserve and function, compromising an individual’s ability to cope with the daily challenges of life [5, 6]. The Frailty Phenotype (FP), which is the most dominant conceptual model in literature [3, 7–10], considers an individual frail by the presence of at least three of five phenotypes: weakness, low levels of physical activity, unintentional weight loss, slow walking speed, and exhaustion. Physical, cognitive, psychological, and social impairments often characterize the different domains of frailty [11]. The physical domain is devoted to FP-related conditions [12], the cognitive domain is the co-existence of physical deficits and mild cognitive impairments [13], the psychological domain focuses on an individual’s coping mechanisms based on their own experiences [14], and the social domain looks at a person’s limited participation in social activities and limitations in social support [15]. Frail older adults are prone to adverse outcomes such as frequent falls, hospitalizations, disabilities, loneliness, cognitive decline, depression, poor quality of life, and even death [16–18]. In response, researchers have proposed various interventions to prevent or slow frailty progression by either targeting a single domain (e.g., physical, social, cognitive, etc.) using single component interventions or targeting two or more domains using multi-component interventions.

For example, Hergott and colleagues investigated the effects of a single-component intervention, functional exercise, on acromegaly-induced frailty [19]. Abizanda and colleagues examined the effects of a multi-component intervention, composed of nutrition and physical activity, on frail older people’s physical function and quality of life [20]. Some studies indicate that certain single or multi-component interventions can either reduce frailty, slow its progression, and possibly reverse it [3, 21, 22]. The current understanding of reverse frailty lacks clarity, and the characteristics of interventions related to frailty reversal have not yet been examined in a systematic manner.

Authors have determined the reversal of frailty using various measures. For instance, Kim and colleagues’ study evaluating an intervention composed of exercise and nutritional supplementation in frail elderly community-dwellers demonstrated reversals in FP components [23]. Components included fatigue, low physical activity, and slow walking, an improvement from the presence of 5 components of frailty (according to the FP) to 2, considered a pre-frail state [23]. Conversely, De Souto and colleagues demonstrated frailty reversal based on changes in frailty index (FI) scores, a measure of accumulation of deficits [24]. A FI score of 0.22 or greater indicates frailty, score less than or equal to 0.10 indicates a non-frail state [25–29]. Hergott et al. (2020) used frailty severity to indicate frailty reversal. Participants in their study reversed frailty from a severe state to a mild state [19]. These studies demonstrate the variability in how reversing frailty is measured and understood. For a more comprehensive understanding of reverse frailty and the characteristics of interventions associated with it, a comprehensive review of the literature on this topic is needed. Therefore, through a scoping review, the aim of this study is to provide an overview and synthesis of interventions that have been implemented for frail older adults, to determine whether some interventions have had an impact on reversing frailty.

This methodology is ideal because it encompasses a broad scope and can comprehensively analyze and synthesize data on a subject [30]. Findings from this review will synthesize the evidence regarding the impact of frail-related interventions on older adults living with frailty, identify what interventions resulted in frailty reversal and clarify the concept of reverse frailty.

### Guiding conceptual framework

The deficit accumulation model framework, unlike the FP, considers frailty as more than a physical deficit but rather an accumulation of health-related deficits across multiple domains [31]. For this reason, the deficit accumulation model framework serves as our guiding conceptual framework. Through this framework, we recognize frailty as a complex phenomenon, strengthening the case for interventions addressing other health and personal concerns, such as illness, environmental disturbance, social dysfunction, cognitive decline, and psychosocial distress. This framework provides a helpful lens through which we can examine the number of domains addressed in the reported interventions and their relationship to one another.

## Methods

We followed Arksey and O’Malley’s [30] five-stage approach, elaborated by Levac et al., [32] and Joanna Briggs Institute (JBI) for scoping review [33]. They propose six stages: (1) identifying the research question, (2) locating relevant studies, (3) selecting the study, (4) charting data, (5) summarizing results, and (6) consulting with stakeholders. We followed the Preferred Reporting Items for Systematic Reviews and Meta-Analyzes Extension for Scoping Reviews (PRISMA-ScR) checklist [34] to guide study reporting. Refer to Additional file 1.

### Stage one: identifying the research question

According to Levac and colleagues, fundamental research questions should be broad enough to enable comprehensive analysis and appropriate mapping of relevant literature [32]. Following this, our three research questions are as follows:

1. What is the available literature on the impact of interventions for frail older adults?
2. Did any of these interventions result in frailty reversal?
3. What does it mean to reverse frailty?

### Stage two: identifying relevant studies

Using the research questions as a guide, we engaged in an iterative process that involved searching the literature, identifying search terms, developing, and refining search strategies, to identify appropriate studies. We also sought the assistance of an experienced librarian who gave guidance on the use of various electronic databases, provided validation on the appropriateness of the methodology for this study, and conducted a peer-review of the search strategies. An overview of each step is provided below.

### Eligibility criteria

JBI’s PCC mnemonic guided eligibility criteria, where P (population): frail older people over 65yrs of age, C (concept): frailty outcome, and C (context): all contexts. We included French and English studies of frail older adults over 65 years because most studies focused on frailty target this age group [35–38]. All types of interventions for frail older adults were included, except for interventions intended to prevent frailty. We did not apply any limitations to study dates, and settings. All study designs (quantitative, qualitative, and mixed methods) were considered for inclusion. We excluded conference abstracts, theses, dissertations, and knowledge syntheses, but did refer to their reference list for potential studies. Lastly, we performed a grey literature scan to identify relevant primary studies to ensure a comprehensive literature search.

### Search terms

An a priori concept analysis [39] of frailty and frailty interventions revealed relevant search terms regarding the population of interest which included ‘frail elderly, frail, aged hospital patient, institutionalized elderly, very elderly, geriatrics, senior, and aged’. These keywords were presented to and approved by an academic librarian (VL). To capture a comprehensive list of studies that may be relevant, we looked at all types of interventions on frail older adults aimed at either reducing, improving, managing, enhancing, treating, or reversing frailty. Medical Subject Headings (MeSH) and boolean operators of these terms were used in different databases to identify relevant studies.

### Search strategy

**Fig. 1:**
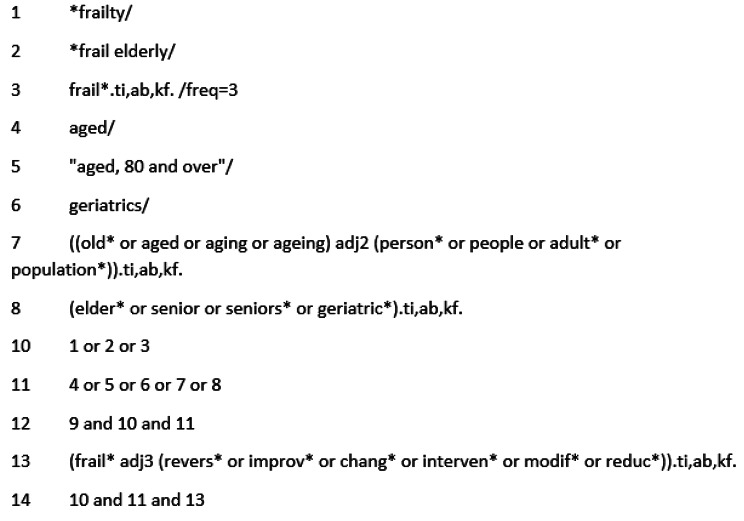
Ovid MEDLINE search strategy

Two academic librarians (VL & VC) guided the development of the search strategy and selected databases. We conducted the searches between August 6th and August 9th, 2021, using MEDLINE (OVID interface), Embase (OVID interface), Cumulative Index to Nursing and Allied Health Literature (CINAHL), and Web of Science. We first implemented the search in MEDLINE (Fig. 1), which we later adapted for the other three databases. We manually searched for relevant studies from the reference lists of included/eligible articles and reviewed conference abstracts and secondary analyzes to identify primary studies. A third academic librarian (LS) peer-reviewed the search strategy using the Peer Review of Electronic Search Strategies (PRESS) guidelines [40] on August 19th, 2021, without modification. On August 23rd, 2021, we imported the results in RIS format into Covidence, a web-based system for systematic reviews provided by Cochrane [41, 42], which also removed duplicates. We did not import the articles identified via hand-searching the reference list into Covidence for screening. However, two reviewers independently assessed the articles’ eligibility according to our eligibility criteria.

### Stage three: study selection

There were two reviewers (AK, OB) involved in this stage, which involved a first and second screening level. The first level included an independent screening of the titles and abstracts, and we decided by selecting ‘yes’, ‘no’, or ‘maybe’. To qualify for full-text screening, a study must receive two ‘yes’ or two ‘maybe’ votes. Two ‘no’ votes moved the study to exclude, and one ‘no’ vote along with one ‘yes’ or ‘maybe’ vote moved it to conflicts, pending resolution. After consultation with the second reviewer, the first author (AK) and second reviewer (OB) resolved the conflicts together. Following this first-level screen, the second level involved a full-text review of all studies included at the title-abstract level. Using the same principles as the first level screening, the first author (AK) and another reviewer (MA) completed this stage [41, 42]. In cases where full-text articles could not be located or had to be purchased, the corresponding authors were contacted once by email to request copies. We excluded the articles if we did not receive a response after two weeks. We also searched Google Scholar for conference abstracts to see if the full text of the papers had been published and accessible. For most searches, this process was ineffective, leading to the exclusion of all conference abstracts. Articles excluded with reasons can be found in Additional file 2.

### Stage Four: charting the data

To extract essential information from the articles, we developed a standard Microsoft Excel form a priori. We used the Template for Intervention Description and Replication (TIDieR) checklist [43] to guide the extraction of the interventions. The form was pilot tested with five articles and revised following recommendations from the research team. After establishing the information to be extracted, we imported the data into Google Forms to facilitate the extracting process for the reviewers. To ensure consistency and reliability in data extraction, two reviewers (AK and MA) independently extracted data from at least 10% of the included studies and compared the results, as recommended by Levac and colleagues [32]. Once we established consistency, the first author (AK) extracted data from the remaining studies.

### Data extracted

Data extraction items include a bibliography (authors, the journal-title and year of publication), setting, study population (frail, number and age of participants), aims of the study, the conceptual framework of frailty used, domains of frailty considered, details on interventions that reduce, enhance, treat or reverse frailty, the framework used to develop interventions, assessment tools or instruments to assess frailty outcome before and/or after the intervention, outcomes (frailty completely, partially, or not reversed). Data extraction items can be found in Additional file 3.

### Quality appraisal (QA)

We critically appraised included studies strengths and limitations of the studies (e.g., randomized controlled trials, quasi-experimental studies, case reports, case series, and cohort studies) using the corresponding JBI checklist for quality appraisal. Checklists, ranged from eight to 13 items [35]. Answers to the questions in each scale ranged from ‘yes’, ‘no’, and ‘unclear’. Three reviewers (YA, MA, and AK) independently appraised the included studies. After completing the assessment, the first author (AK) sorted the answers to determine any discrepancies. When two reviewers reported the same answer, agreement was achieved. When answers differed, the first author extensively reviewed the study and discussed the differences with the other two to reach a consensus. After completion, we converted all the answers into descriptive variables, with yes representing ‘1’ and no and unclear meaning ‘0’. Following recommendations from some studies [44, 45], we used these variables to generate a total score, which we further used to classify a study into “low”, “moderate”, and “high” risk of bias. The quality appraisal interpretation scale can be found in Additional file 4.

### Stage five: summarizing and reporting the results

#### Data analysis

To summarize and elaborate on the first research question, we used a narrative synthesis. Initially, we developed a preliminary synthesis by grouping studies that focused on similar concepts such as but not limited to types of interventions, domains of frailty targeted, outcome of interventions, into a tabular format. Next, using excel, we created bar graphs where we explored relationships between and within studies. Through the use of conceptual mapping, we linked multiple pieces of evidence from individual studies to highlight key concepts and ideas [46, 47].

Our approach to answering the second research question, comparing study demographics and participant characteristics, was descriptive in nature. Using Excel, we calculated the counts and frequencies of variables in each category and compared their percentages across studies [48].

## Results

### Study selection

**Fig. 2:**
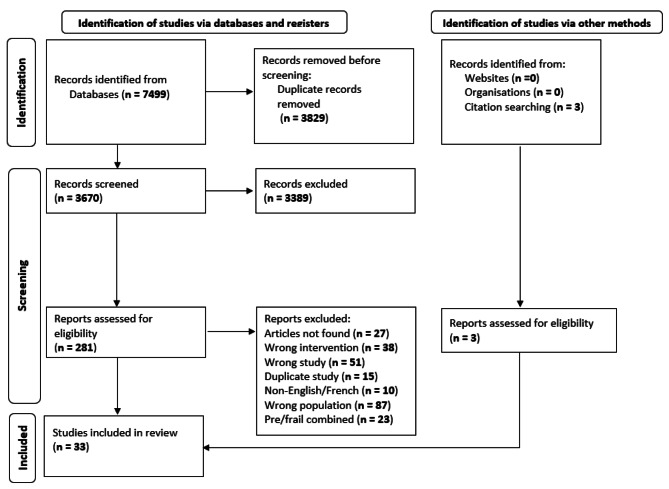
PRISMA flow diagram of the search process for studies

We identified 7499 potential records, of which thirty met eligibility criteria. In addition, our hand search of references of included studies revealed three eligible studies, reaching a total of thirty-three. We illustrate the screening and selection process for the included studies using the PRISMA 2020 flow diagram for systematic reviews (Fig. 2).

### Study characteristics

Sample sizes ranged from one to 250,428 participants across the studies. The most common study designs were randomized controlled trials (RCTs) (n = 23) [22–24, 49–68], quasi-experimental (n = 4) [69–72], cohort Studies (n = 3) [20, 73, 74], case series (n = 2) [75, 76] and a case report (n = 1) [19]. Geographically, the studies took place in fifteen different countries, namely Japan (n = 6) [23, 49, 53, 58, 72, 74], Spain (n = 6) [20, 59, 60, 62, 70, 75], United States of America (n = 4) [19, 63, 64, 68], China (n = 3) [51, 52, 69], Sweden (n = 2) [50, 55], South Korea (n = 2) [71, 76], Singapore (n = 2) [22, 54], Australia (n = 1) [66], Netherlands (n = 1) [65], Canada (n = 1) [73], France (n = 1) [24], Brazil (n = 1) [67], Thailand (n = 1) [56], Turkey (n = 1) [57], Denmark (n = 1) [61]. Publication dates ranged from June 23rd, 1994, to January 2nd, 2021, with most articles (n = 24) published after 2015.

### Critical appraisal results

The quality assessment scores of the studies ranged from seven to twelve, and study bias was low to moderate for all included studies (Appendix 4). Given that scoping reviews do not mandate the inclusion of studies based on critical appraisal results [77], we did not exclude studies based on their quality assessment cores.

### Participant characteristics

Twelve studies (36.4%) included participants over 65 years of age, 11 studies (33.3%) over 70 years of age, and 10 studies (30.3%) over 75 years of age. Most authors referred to participants as male or female without definition making it difficult to distinguish between gender and sex. Consequently, we present the results as reported in the studies. All but one study reported the sex/gender of participants [57], with one study having only male participants [19] and two studies having only female participants as per their eligibility criteria [23, 61]. In many studies, the presence of comorbidities beyond frailty was not a requirement for participation (n = 27). Some studies, however, required comorbid conditions for inclusion, such as acromegaly (n = 1) [19], cardiovascular disease (n = 1) [72], chronic obstructive pulmonary disease/lung disease (n = 1) [60], fatigue (n = 1) [69], and risk of mobility disability and sedentary lifestyle (n = 1) [64]. Table 1 presents a summary of participant characteristics.

**Table 1:** Study and participant characteristics

Author/year	Research design	Country of study	Age (years)	Comorbidity	Number of participants		Female (%)	Setting	Critical Appraisal Rating
					Control group	Intervention group			
Abizanda, 2015 [20]	Cohort Studies	Spain	≥ 70	NR	0	69	70	Nursing/ Retirement	Moderate
Arrieta, 2019 [62]	RCT	Spain	≥ 70	NR	45	43	70.5	Nursing/ Retirement	Low
Brown, 2000 [63]	RCT	USA	≥ 75	NR	36	48	56	Nursing/ Retirement	Moderate
Cadore, 2014 [75]	Case Series	Spain	≥ 75	Dementia	NR	11	55	Nursing/ Retirement	Low
Cameron, 2013 [66]	RCT	Australia	≥ 70	NR	109	107	68	Community-Dwelling	Moderate
Cesari, 2015 [64]	RCT	USA	≥ 70	Risk of mobility disability, Sedentary lifestyle.	211	213	68.9	Community-Dwelling	Moderate
Chin A Paw, 2001 [65]	RCT	Netherlands	≥ 70	NR	37	120	70	Community-Dwelling	Moderate
Coelho-Júnior, 2021 [67]	RCT	Brazil	≥ 65	NR	13	26	64.3	Community-Dwelling	Moderate
de Souto Barreto, 2018 [24]	RCT	France	≥ 70	NR	163	185	64.7	Community-Dwelling	Low
Fiatarone, 1994 [68]	RCT	USA	≥ 70	NR	26	74	63	Nursing/ Retirement	Moderate
Hergott, 2020 [19]	Case report	USA	≥ 70	Acromegaly	NR	NR	0	Nursing/ Retirement	Moderate
Imaoka, 2016 [49]	RCT	Japan	≥ 75	NR	17	58	75.8	Nursing/ Retirement	Moderate
Kim, 2015 [23]	RCT	Japan	≥ 75	NR	33	98	100	Community-Dwelling	Moderate
Kim, 2020 [76]	Case Series	South Korea	≥ 70	NR	NR	10	100	Community-Dwelling	Low
Lammes, 2012 [50]	RCT	Sweden	≥ 75	NR	21	72	60	Community-Dwelling	Moderate
Larsen, 2020 [73]	Cohort Studies	Canada	≥ 65	NR	NR	110,297	57.6	Community-Dwelling	Low
Li ,2010 [51]	RCT	China	≥ 65	NR	31	26	47.7	Community-Dwelling	Low
Liao, 2019 [52]	RCT	China	≥ 70	NR	16	16	69.2	Community-Dwelling	Low
Liu, 2017 [69]	Quasi-Experimental	China	≥ 65	Fatigue	21	58	92	Community-Dwelling	Low
Losa-Reyna, 2019 [70]	Quasi-Experimental	Spain	≥ 75	NR	5	8	75	Community-Dwelling	Low
Nagai, 2018 [53]	RCT	Japan	≥ 65	NR	9	7	90	Community-Dwelling	Moderate
Ng, 2015 [22]	RCT	Singapore	≥ 65	NR	7	61	61.4	Community-Dwelling	Low
Ng, 2017 [54]	RCT	Singapore	≥ 65	NR	7	61	61.4	Community-Dwelling	Low
Oh, 2021 [71]	Quasi-Experimental	South Korea	≥ 65	NR	196	187	72.3	Community-Dwelling	Low
Rydwik, 2010 [55]	RCT	Sweden	≥ 75	NR	14	50	60	Community-Dwelling	Moderate
Sadjapong, 2020 [56]	RCT	Thailand	≥ 75	NR	32	32	61	Community-Dwelling	Low
Sahin, 2018 [57]	RCT	Turkey	≥ 65	NR	16	32	NR	Nursing/ Retirement	Low
Seino, 2017 [58]	RCT	Japan	≥ 65	NR	0	21	31	Community-Dwelling	Low
Takatori, 2021 [74]	Cohort Studies	Japan	≥ 75	NR	NR	942	50.3	Community-Dwelling	Low
Tarazona-Santabalbina, 2016 [59]	RCT	Spain	≥ 70	NR	49	51	54	Community-Dwelling	Low
Torres-Sánchez, 2017 [60]	RCT	Spain	≥ 65	COPD/Lung disease	29	29	27.6	Hospital-Based	Low
Ushijima, 2021 [72]	Quasi-Experimental	Japan	≥ 65	Cardiovascular disease	66 (non-frail group)	23 (frail group)	23.6	Hospital-Based	Low
Vestergaard, 2008 [61]	RCT	Denmark	≥ 75	NR	28	25	100	Community-Dwelling	Moderate

NR = Not reported

### Most and least common domains targeted

**Fig. 3:**
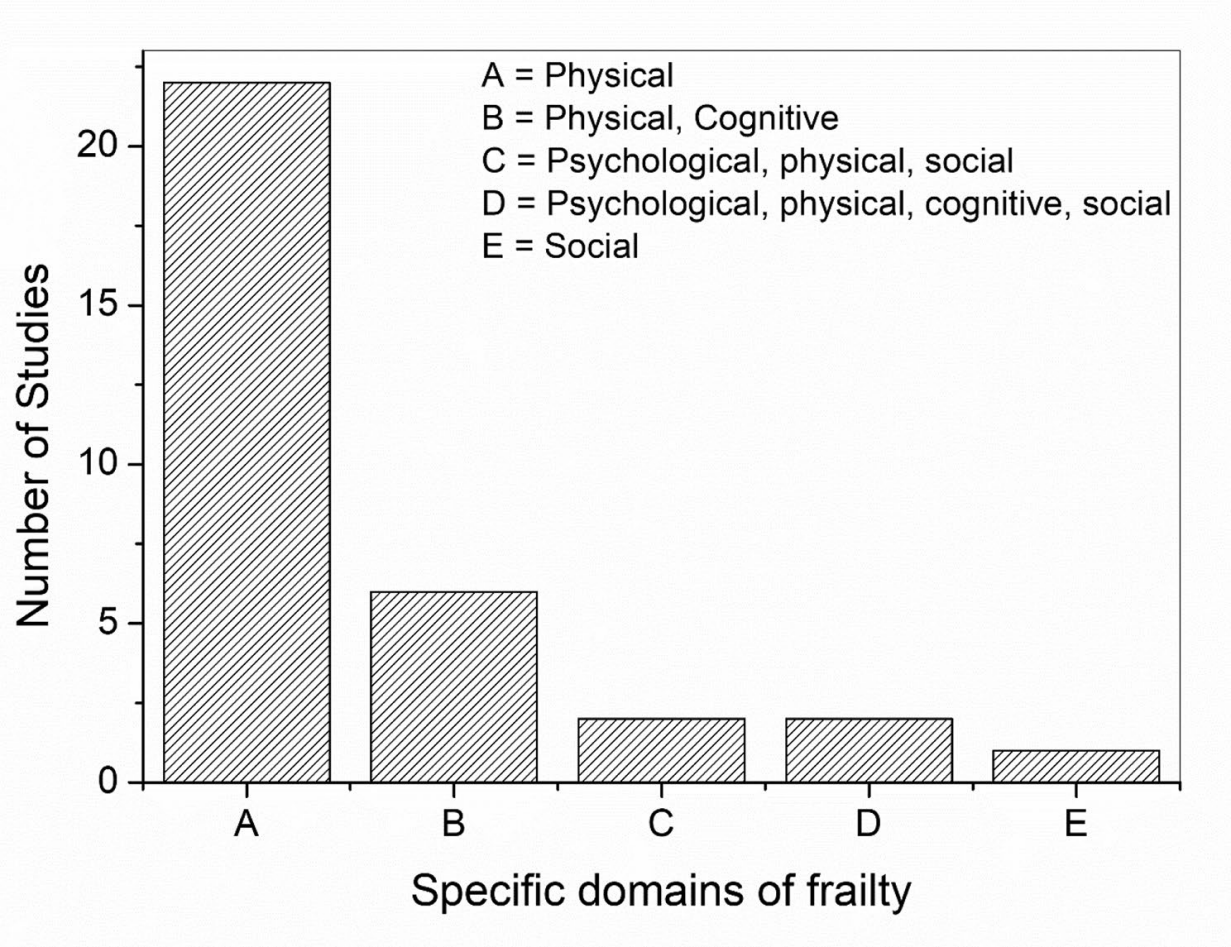
Breakdown of the domains identified in studies

Twenty-six studies involved intervention and control groups. Additionally, each study’s intervention targeted at least one domain of frailty. For example, some interventions targeted one single domain (n = 23) [19, 20, 23, 49, 50, 52, 53, 55–57, 59–65, 67, 68, 70, 72–74], two domains (n = 6) [4, 22, 54, 56, 57, 78], three domains (n = 2) [58, 66], and four domains of frailty (n = 2) [51, 71]. Counts per domain are presented in Fig. 3. The most targeted domains were the physical and the cognitive domains. The social domain was the least targeted.

### Single and multi-component interventions

Thirteen studies (39.4%) focused on single-component interventions; twelve were physical activity interventions [52, 53, 56, 60, 62–64, 67, 70, 73, 76], and one was a social intervention [74]. These activities were either individually tailored or performed in a group. Over 50% of the studies focused on multicomponent interventions [19, 20, 22–24, 49–51, 54, 55, 58, 59, 65, 66, 68, 69, 71, 72, 75]. The number of components varied across interventions; from two components to the interventions (n = 10) [20, 23, 49, 50, 55, 59, 65, 68, 69, 75], three components to the interventions (n = 8) [19, 22, 24, 54, 58, 66, 71, 72], or four components to the interventions (n = 2) [51, 71]. Characteristics of the interventions are.

included in Table 2.

**Table 2:** Study intervention characteristics

Author (year) Study country	Study objective	Frailty Framework	Frailty Domains Targeted	Characteristics of interventions	-Frequency -Duration -PIFU	Frailty Reversed?	Outcome of intervention
SINGLE-COMPONENT INTERVENTIONS							
Arrieta, 2019 [62] **Spain**	To determine the general effect of the intervention on frailty outcome.	Fried	Physical	Physical activity Balance, strength, and walking exercises at progressive to moderate intensity.	**Freq =** 2x/wk **Dur =** 26wks **PIFU =** 6 m	No	Lower prevalence of frailty
Brown, 2000 [63] **USA**	To determine the general effect of the intervention on physical frailty.	PPT ≤ 32	Physical	Physical activity Strength, Balance, Sensation, Coordination and range of motion	**Freq =** 3x/wk **Dur =** 12wks **PIFU =** NR	No	Decreased prevalence of frailty, increased physical performance test scores.
Cesari, 2015 [64] **USA**	To determine the effect of physical activity in reducing frailty prevalence.	Fried	Physical	Physical activity Walking, Aerobic, Strength, Flexibility, and balance are all done at progressive intensities.	**Freq=** Weekly 1-2x/wk 3x/wk **Dur =** 52wks **PIFU =** NR	No	Frailty prevalence reduced.
Coelho-Júnior, 2021 [67] **Brazil**	To determine the effects of the intervention on frailty status	Fried	Physical	Physical activity Resistance exercise at progressive to moderate intensity.	**Freq =** Daily **Dur =** 16wks **PIFU =** NR	Yes	
Kim, 2020 [76] **South Korea**	To determine the effectiveness of interventions on cognitive and depressive functions.	Fried	Physical, Cognitive	Physical activity Aerobic, Strength, Flexibility. Exercise intensity not reported.	**Freq =** Monthly **Dur =** 12wks **PIFU =** NR	No	Improved mental state and cognitive function.
Larsen, 2020 [73] **Canada**	To determine the effects of the intervention on frailty status	Frailty index	Physical	Physical activity Activity characteristics not reported	NR	Yes	
Liao, 2019 [52] **China**	To determine the effectiveness of interventions on frailty status.	Fried	Physical	Physical activity Resistance, aerobic, balance, exergaming at progressive intensities.	**Freq =** 3x/wk **Dur =** 12wks **PIFU =** NR	Yes	
Losa-Reyna, 2019 [70] **Spain**	To determine the effect of the intervention on physical and frailty outcomes.	Fried	Physical	Physical activity Resistance, Strength, Aerobic performed at high intensity.	**Freq =** 2x/wk **Dur =** 6wks **PIFU =** NR	Yes	
Nagai, 2018 [53] **Japan**	To determine the effect of the intervention on the physical and mental outcomes of frail individuals	Fried	Physical	Physical activity Resistance at progressive intensity	**Freq =** 2x/wk **Dur =** 24wks **PIFU =** NR	No	Increased physical strength, decreased frailty score, no significance in frailty status.
Sadjapong, 2020 [56] **Thailand**	To determine the effect of intervention in reversing frailty and functional outcome	Fried	Physical	Physical activity Aerobic, balance, resistance at progressive moderate to high intensity.	**Freq =** 3x/wk **Dur =** 24wks **PIFU =** NR	Yes	
Sahin, 2018 [57] **Turkey**	To determine the effectiveness of the intervention on functional outcome	Fried	Physical	Physical activity Balance exercise at low and high intensities.	**Freq =** 3x/wk **Dur =** 8wks **PIFU =** NR	No	Increased SPPB score (better in HI group), Increased Barthel index score, decreased fatigue severity, increased muscle strength.
Takatori, 2021 [74] **Japan**	To determine the effect of the intervention on frailty reversal.	Kihon checklist	Social	Social activity Enhance social capital, such as interaction with neighbours, trust in the community, etc.	NR	Yes	
Torres-Sánchez, 2017 [60] **Spain**	To determine the effect of the intervention on disability	Frailty index	physical	Physical activity Pedal exercise	**Freq =** NR **Dur** = Based on length of stay at the institution **PIFU =** NR	No	Increased exercise capacity, increased muscle strength, increased balance
Vestergaard, 2008 [61] **Denmark**	To determine the effect of the intervention on functional outcome	NR	Physical	Physical activity Aerobic, Flexibility, Balance, Strength done at progressive intensities	**Freq =** 3x/wk **Dur =** 20wks **PIFU =** NR	No	Improved functional outcomes such as walking speed, biceps & handgrip strength, balance, and mobility.
**MULTICOMPONENT INTERVENTIONS**							
Abizanda, 2015 [20] **Spain**	To determine the effect of intervention in improving functionality	Fried	Physical	Physical activity + Nutrition **Exercise =** Flexibility, balance, strength. Intensity not reported. **Nutrition =** Supplements	**Freq =** 5x/wk **Dur =** 3yrs **PIFU =** NR	No	Increased nutritional and functional status
Cadore, 2014 [75] **Spain**	To determine the effect of the intervention on falls and functional outcome	Fried	Physical Cognitive	Physical activity + Cognitive **Exercise =** Walking, Balance, Resistance. Intensity not reported **Cognitive =** cognitive exercise	**Freq =** 2x/wk **Dur =** 24wks **PIFU =** 6 m	No	No significance on any of the physical outcomes assessed.
Cameron, 2013 [66] **Australia**	To determine the effectiveness of intervention in reducing frailty and improving mobility	Fried	Physical Psychological Social	Physical activity + nutrition + social intervention **Exercise =** Balance exercise at moderate intensity **Nutrition =** Home delivered meals, supplements **Social =** increased access to social interactions	**Freq =** 3-5x/wk **Dur =** NR **PIFU =** NR	No	Decreased frailty score by 0.80, Increased SPPB
Chin A Paw, 2001 [65] **Netherlands**	To determine the effect of the intervention on functional outcome	Chin a Paw	Physical	Physical activity + Nutrition **Exercise =** Strength, Flexibility, and Endurance done progressively. **Nutrition =** supplements	**Freq =** 2x/wk **Dur =** 26wks **PIFU =** NR	No	Slight increase in ADL score, increased fitness and balance, no effect on disability score
de Souto Barreto, 2018 [24] **France**	To determine the association of intervention with frailty severity.	Frailty index	Physical Cognitive	Physical activity + Nutrition + Cognitive **Exercise =** Advice on varying exercises **Nutrition =** counseling **Cognitive =** Memory and reasoning training	**Freq =** Monthly **Dur =** 24wks **PIFU =** NR	No	Though no significant effect on frailty severity, it has a higher chance of reducing frailty development.
Fiatarone, 1994 [68] **USA**	To determine the effect of intervention in reducing frailty	NR	Physical	Physical activity + Nutrition **Exercise =** Resistance exercise done progressively at high intensity. **Nutrition =** supplements	**Freq =** 3x/wk **Dur =** 10wks **PIFU =** NR	No	Increased physical activity, increased muscle strength, increased gait velocity.
Hergott, 2020 [19] **USA**	To determine the effect of intervention in reversing frailty	CFS	Physical	Physical activity + occupational therapy + speech therapy **Physical activity =** Types of activities not identified. Exercises done at progressive intensities. **occupational therapy =** No further details presented **Speech therapy =** No further details presented	**Freq =** 12 h/week **Dur =** 17 wks **PIFU =** NR	Yes	
Imaoka, 2016 [49] **Japan**	To determine the effect of the intervention on falls	NR	Physical	Physical activity + Nutrition **Exercise =** Resistance, Balance, Strength at low frequencies. Intensities not reported. **Nutrition =** supplements	**Freq =** 2x & 3x weekly **Dur =** 24wks **PIFU =** 6 m	No	Rate of falls decreased by 72.4%
Kim, 2015 [23] **Japan**	To determine the effect of the intervention on frailty status	Fried	Physical	Physical activity + Nutrition **Exercise =** Resistance, Balance, Strength, and Gait training at progressive intensities **Nutrition =** Supplements	**Freq =** 2x/wk **Dur =** 8wks (4 + 4 wks) **PIFU =** 1 m	Yes	
Lammes, 2012 [50] **Sweden**	To determine the effect of the intervention on body composition.	Chin a Paw	Physical	Physical activity + Nutrition **Exercise =** Aerobic, Strength, Balance progressively at low-intensity. **Nutrition =** Counseling	**Freq =** 2x/ wk **Dur =** 52wks **PIFU =** 6 m	No	No effect on body composition, no effect on energy intake, Increased RMR.
Li ,2010 [51] **China**	To determine the effect of the intervention on frailty status	Fried	Physical Social Psychological Cognitive	Activities designed per participant’s need.	Specific to participant’s needs	No	No significant outcome on frailty status and Barthel index.
Liu, 2017 [69] **China**	To determine the general effect of the intervention	Fried	Physical Psychological	Physical activity + Behavioral intervention **Exercise =** Aerobic, Balance, Resistance at progressive intensities **Behavioral =** Motivational enhancement	**Freq =** weekly **Dur =** NR **PIFU =** NR	No	Increased physical endurance, increased rate of participation in activities.
Ng, 2015 [22] **Singapore**	To determine the effect of intervention in reversing frailty	Fried	Physical Cognitive	Physical activity + Nutrition + cognitive **Exercise =** Resistance, Balance at increasing progressive intensities **Nutrition =** supplements **Cognitive =** Stimulate short-term memory, enhance attention, information processing, reasoning, and problem-solving skills	**Freq =** 2x/wk **Dur =** 12wks **PIFU =** 6 m	Yes	
Ng, 2017 [54] **Singapore**	To determine the effect of the intervention on depressive symptoms	Fried	Physical Cognitive	Physical activity + Nutrition + cognitive **Exercise =** Resistance, Balance at increasing progressive intensities **Nutrition =** supplements **Cognitive =** Stimulate short-term memory, enhance attention, information processing, reasoning, and problem-solving skills	**Freq =** 2x/wk **Dur =** 12wks **PIFU =** 6 m	No	No significance on GDS
Oh, 2021 [71] **South Korea**	To determine the general effect of the intervention	Fried + Frailty index	Physical Social Cognitive Psychological	Physical activity + Nutrition + Social + Pharmaceutical + Psychological **Exercise =** Resistance, Balance, Aerobic at progressive intensities **Nutrition =** supplements **Social =** Increase access to social interactions **Psychological =** psychotherapy **Pharmaceutical =** deprescription and depression management	**Freq =** 2x/wk **Dur =** 12 wks **PIFU =** 18 m & 30 m	No	Lower frailty phenotype & frailty index scores. No statistically significant difference in CES-D score. No statistically significant prevalence of polypharmacy. Lower rates of mortality. Lower rates of long-term care institutionalization.
Rydwik, 2010 [55] **Sweden**	To determine the effect of the intervention on functional outcome	Chin a Paw	Physical	Physical activity + Nutrition **Exercise =** Aerobic, Strength, Balance progressively at moderate intensity. **Nutrition =** Counseling + supplements	**Freq =** 2x/wk **Dur =** 52wks **PIFU =** 2yrs	No	No significant changes in physical activity. No effects on ADLs
Seino, 2017 [58] **Japan**	To determine the effect of the intervention on frailty status	Check-List 15	Physical, Psychological, Social	Physical + Nutrition + Social intervention **Exercise =** Resistance exercise at progressive intensity. **Nutrition =** counseling **Social =** Improve social skills, increase social support, and increase access to social interactions & community gatherings. Structured counselling, relapse prevention.	**Freq =** 1x q 2 wks **Dur =** 12wks **PIFU =** NR	No	Decreased prevalence of frailty, increased social participation
Tarazona-Santabalbina, 2016 [59] **Spain**	To determine the effect of intervention in reversing frailty and improving functional outcome	Fried	Physical	Physical activity + Nutrition. **Exercise =** Proprioception, Aerobic, Strength, Stretching **Nutrition =** Counseling	**Freq =** 5x/wk **Dur =** 24wks **PIFU =** NR	Yes	
Ushijima, 2021 [72] **Japan**	To determine the effect of the intervention on functional outcome	Fried	Physical	Physical activity + Nutrition + Pharmaceutical **Exercise =** Resistance, aerobic at individually tailored intensities **Nutrition =** counseling **Pharmaceutical =** Medication guidance, CPR resuscitation practice	**Freq =** 3-5x/wk **Dur =** 12 wks **PIFU =** 30 m	Yes	

**Notes: PIFU** (Post-intervention follow-up); **Freq** (Frequency); **Dur** (Duration); **NR** (Not reported); **RMR** (Resting metabolic rate); **ADLs** (Activities of daily living); **iADL** (Instrumental Activities of Daily Living); **CES-D** (Center for Epidemiologic Studies Depression Scale); **RCTs** (Randomized control trials); **COPD** (Chronic obstructive pulmonary disease); **BMI** (Body Mass Index); **RT** (Resistance training); **CPR** (Cardiopulmonary resuscitation); **CFS** (Clinical Frailty Scale); **wks** (weeks); **yrs** (years); **m** (months); **≤** (Less than or equal to); **PPT** (Physical Performance Test; **q** (every)

### Most and least common frailty definitions used

Frailty was defined in all but three studies (n = 30) [49, 61, 68]. Two different definitions of frailty were used dominantly: Fried’s phenotype (n = 20) [20, 22, 23, 51–54, 56, 57, 59, 62, 64, 66, 67, 69–72, 75, 76], and the Frailty Index (n = 4) [24, 60, 71, 73]. Notwithstanding, other definitions of frailty involved the use of the clinical frailty scale [19] and checklist such as the kihon checklist [74].

### Studies without frailty reversal outcome

In the 33 studies included, the results of 22 did not indicate reversal of frailty. Among these, 36.36% (n = 8) focused solely on physical interventions [53, 57, 60–64, 76], while 63.63% (n = 14) combined physical activity with nutritional, cognitive, social, pharmaceutical, or behavioral interventions [20, 24, 49–51, 54, 55, 58, 65, 66, 68, 69, 71, 75]. Although physical activity remains a significant factor in these studies, the types of physical activity (aerobic, strengthening, gait, resistance, etc.) varied. Research suggests that resistance exercise performed at high intensity over a minimum of 12 weeks has the most beneficial effect on physical frailty [68, 79]. When done regularly over the course of six months, it has the potential to improve both the physical and physiological aspects of frailty [80]. In this context, we noted that resistance exercise was more prevalent than other forms of physical activity. Although similar physical activities were often implemented, their characteristics often differed. For example, there was variation in frequency from daily to three times per week, variation in intensity from moderate to high, and variation in duration from 6 weeks to 6 months.

In addition to physical activity, other types of interventions were also used, including cognitive interventions such as memory and reasoning training, pharmaceutical interventions such as medication reconciliation, social interventions such as improving social lifestyles, and behavioral interventions such as goal setting, action plans, and goal execution. Similarly, the characteristics of these interventions were heterogeneous across studies, with some provided as group therapies, and others designed as per the needs of participants.

### Studies indicating frailty reversal outcome

Eleven studies reported frailty reversal as an outcome [19, 22, 52, 56, 59, 67, 70, 72–74, 81]. The physical domain was targeted in over 80% of the studies (n = 9) [19, 23, 52, 56, 59, 67, 70, 72, 73], while the social [74] and cognitive domains [22] were each targeted in one study. In single-component interventions such as physical activities (n = 5) [52, 56, 67, 70, 73], resistance exercises appeared to be the most common, done on its own or in combination with other physical exercises. Meanwhile, the social intervention enhanced the patient’s social capital, a social network that facilitates access to benefits and helps individuals solve problems through association [74].

The multi-component intervention consisted of physical activity combined with either nutritional counselling/advice or supplements. Some (n = 5) of the interventions included physical activity, nutrition, plus pharmaceutical intervention in one study [72], physical activity, nutritional plus cognitive intervention in another study [22], and physical activity combined with occupational and speech therapy [19], with intervention characteristics varying across studies.

### Definition/clarity about the concept of reverse frailty

Authors of 17 studies referred to frailty as a reversible condition. However, the concept of reversing frailty was not defined or explained in six studies [22, 54, 57, 58, 63, 64]. When defined, definitions varied. Some authors defined it as a shift from a frail to pre-frail state (n = 1) [56], frail to non-frail (n = 2) [24, 59], frail to pre- and non-frail (7) [23, 52, 67, 70, 72–74], and severe frailty to mild frailty (n = 1) [19]. What was common across all definitions is that the direction of reversal was from a more severe state of frailty to a less severe state of frailty or pre-frail state. What is different is the degree of frailty, given that some definitions indicated a participant should be frail while others indicated participants being severely frail. This suggests the use of different definitions, criteria, methods, and measures to determine whether frailty reversal occurred. For example, seven of the studies that showed reversal used the definition of Fried et al., [23, 52, 56, 59, 67, 70, 72], one study used the frailty index [73], and another study used the clinical frailty scale [19]. Finally, one study used the Kihon checklist, consisting of 25 yes or no questions on daily-life-related activities, motor functions, nutritional status, oral functions, homebound, cognitive functions, and depressed mood [74].

## Discussion

Our study aimed to summarize and synthesize evidence on the impact of interventions on frail older adults, to identify those that resulted in frailty reversal and those that did not. In cases where frailty reversal was indicated, we explored the meaning of the concept of reversing frailty. Among the 33 studies included, frailty was revealed to be a complex syndrome encompassing multiple domains, indicating the need for interventions targeting different aspects. Even though some interventions were more prevalent, we observed similarities between types of interventions across studies that showed frailty reversal and those that did not. We noted that the physical domain received the most attention across all studies, whereas the social domain received the least attention in studies with frailty reversal outcomes. Considering that frailty has been defined, addressed, or assessed in multiple ways throughout the studies, further exploration will contribute to clarifying the concept of reversing frailty. These findings lead us to the following points.

### Frailty reversal may depend on targeted domains

To the best of our knowledge, the present study is the first to systematically map interventions that indicate frailty reversal as an outcome and relates these interventions to the targeted frailty domains. Using the deficit accumulation model framework as our conceptual framework, we anticipated interventions would target multiple domains of frailty to achieve frailty reversal. However, this was not the case. We identified that the physical domain of frailty is the most frequently targeted as compared to the cognitive, social, and psychological domains. This is supported by the findings of other reviews where authors perceived frailty as primarily a physical impairment, measured by the Fried criteria [82–87]. This finding suggests that reversing frailty may probably depend on the domain that is targeted by the intervention, or the conceptual framework used to identify and measure its outcome.

### Definition of reverse frailty remains unclear

There is no standard definition of reverse frailty, yet the concept appears in several research studies. We used a descriptive approach such as percentages to examine the differences and similarities between the various definitions. A fundamental similarity is that the individual must be deemed frail at baseline. However, the process of determining an individual’s frailty score or status differed among the studies because of the different assessment instruments used. Another similarity was that to reverse frailty, frailty scores or status must not progress to a more severe state but rather improve to a pre-frail or milder state of frailty. Further research is required to clarify this concept, preferably through concept analysis.

### Absence of a universal method to reverse frailty

This review included a heterogeneous group of studies with a diverse range of participant characteristics, intervention types, and duration of intervention. Single-component and multi-component interventions have shown efficacy in reversing frailty, with more studies of single-component interventions (i.e., physical activity or social interventions) than the latter.

### Use of single-component interventions to reverse frailty

Our study identified physical activity as the most used intervention across studies that reversed frailty. This fits with previous findings that physical activity is essential in interventions for frail older adults [85–88]. The activities were performed together (combination exercises) or separately (resistance only). In one study, frailty was reversed as early as six weeks [70]. The authors attributed this to the combination of resistance, strength training and aerobic exercises. Therefore, when combined with other types of exercise, resistance exercise could promote the rapid improvement of physical frailty.

According to a recent scoping review, social frailty has not received adequate attention [15]. Based on the findings of our review, we agree with this notion, given we identified only one study [74] that explored frailty reversal through singular intervention. Using an established checklist of items, the study monitored the effects of enhanced social capital (including interaction with neighbours, trust in the community, social participation in activities) on frailty reversal over two years. The results showed that 31.8% of the participants’ frailty statuses reversed to pre-frail or non-frail Another study [58] showed that increasing participants’ social capital improved their adherence to activities and encouraged them to continue interventions even after the study had ended. Thus, interventions that consider this approach may have better outcomes when it comes to frailty reversal.

### Use of multi-component interventions to reverse frailty

The studies(n = 11) that showed frailty reversal as an outcome employed a combination of two or more intervention components tailored to participant needs or conducted in small groups. Physical activity, particularly resistance exercise, is recommended in conjunction with nutritional interventions as a preventative measure of muscle atrophy in older adults [58], which may explain why this combination was the most common among the multi-component interventions. We also noted other physical activities such as strength, balance gait and aerobic exercise performed in combination with resistance exercise at varying frequencies and durations. Nutritional interventions included dietary supplements and nutritional education (advice and counselling) on healthy food choices, with the latter being the most reportedly used. We related the advantage of this approach as reported in other studies where Interventions that aimed to empower participants by way of soliciting and incorporating their input (e.g., choosing meals) were more likely to result in participants feeling in control and autonomous over their dietary choices [89, 90]. This may explain how nutritional education may provide older adults with more food variety and improved food intake compared with dietary supplements [58]. In addition to nutritional education and physical activity, Ushijima et al. [72] also provided medication guidance, to mitigate the effects of polypharmacy, which have been shown to negate the effects of physical and nutritional interventions [91, 92].

### Recommendations

The results and discussion points above guide our research, practice, and policy recommendations.

#### Research

In this scoping review, the reporting of the interventions was suboptimal. For example, not all studies reported whether interventions were modified, personalization of interventions were planned, fidelity and adherence were measured, or how intervention fidelity was maintained or improved. Therefore, we recommend that authors use the template for intervention description and replication (TIDIER) checklist [43] or the Standards for Reporting Implementation Studies (StaRI) [93] whenever possible to improve intervention reporting. These checklists facilitate clinician use of interventions and researchers’ synthesis and replication. Additionally, we recommend that authors of future studies provide details on the definition and components of frailty. Clinically, this may help identify groups of individuals in need of care and facilitate understanding among researchers.

Despite having no study design restrictions, we did not identify any qualitative or mixed method studies about frailty reversal interventions. None of the included studies reported engaging participants in decision-making or incorporating participant experiences into intervention delivery. A recent scoping review [94] echoes this concern, as older adults worry that they are not involved in health and well-being decisions. It is known that engaging older adults in decision-making improves health outcomes [95]. Therefore, we recommend qualitative and mixed methods studies aiming to integrate the older adults’ perspective regarding intervention development, evaluation, or implementation.

Acknowledging that frailty is complex in nature, RCTs with a large sample size could be beneficial to investigate the social, psychological, and cognitive aspects of frailty, which have received little attention to date.

Among the studies that did not report frailty reversal as an outcome, behavioural enhancement was one of the interventions implemented. The use of behavioral enhancement has been associated with the development of self-management skills and the maintenance of long-term changes [69]. It is therefore our recommendation that more studies consider a behavioural enhancement approach to facilitate adherence to interventions and maintain the benefits of interventions over the long-term. Lastly, given that frailty assessments and measurements are inconsistent, there is a need for more work to standardize them.

#### Practice

Further to considering the perspectives of older adults with frailty, we recommend tailoring interventions to fit the needs and capabilities of individuals rather than generalizing it across an entire population. For example, Latham and colleagues [96] conducted a resistance training program with Vitamin D supplements over ten weeks for participants with certain functional limitations, such as dependence on others for activities of daily living, prolonged bed rest, or impaired mobility. Contrary to other studies reporting positive effects of resistance exercise, such as improved functional outcomes and decreased frailty scores during this period [53, 58, 67, 68], Latham and colleagues reported increased fatigue and musculoskeletal injury risks, which may be related to the participants’ functional limitations. We, therefore, recommend tailoring interventions to match participants’ needs and abilities rather than having set durations, frequencies, or intensities of interventions. Another reason is that some older adults may have functional limitations affecting their ability to adhere to prescribed interventions, including the potential adverse effects of polypharmacy on intervention effectiveness [92].

#### Policy

Research results influence guidelines and expectations for delivering care, services, and programs [97]. Frailty is becoming a potential public and global health concern, as indicated by the inclusion of studies from North America, Europe, Asia, Australia, etc. This reinforces the need to prevent or reverse this geriatric syndrome. Future studies should investigate frailty in all continents to increase our understanding on the global challenges of expectations, implementation, or care delivery for frail older adults. Such information can facilitate the transfer of healthcare professionals between continents by bridging the knowledge gap concerning frailty, its interventions, and potential strategies for reversing the condition.

### Strengths and limitations

Our study has strengths and limitations. We established a reproducible, systematic approach, from the literature search to screening and data extraction. Furthermore, the search strategy was guided and peer-reviewed by academic librarians with extensive knowledge of scoping and systematic reviews. We quality appraised included articles permitting us to have a better sense of the quality of the evidence on this topic. Although not formally published or registered, an a priori protocol approved by the research team guided this study. In comparison to the protocol, a few changes have been made to this study, such as not obtaining expert consultation and revising the research questions.

In terms of limitations, included studies were heterogeneous in their study objectives, frailty definition, frailty domain targeted, and intervention characteristics. Some studies used self-administered questionnaires as outcome measures to assess frailty, potentially increasing the risk of bias and making replication difficult because there is no guarantee of having the same responses among different participants. In addition, two studies did not report the characteristics of the intervention [19, 73], and one indicated that participants were frail but did not specify how frailty was determined [68]. Lastly, we acknowledge that using only a few databases may have limited the number of studies we were able to find.

## Conclusions

We used a narrative and descriptive approach to synthesize the included studies. Despite the lack of a standard definition of frailty, we observed similar interventions across studies that reported an outcome of frailty reversal and those that did not. When frailty reversal was indicated, we explored the meaning of the concept. We noted that the physical domain received the most attention across all studies. In contrast, the social domain received the least attention in studies with frailty reversal outcomes.

This study confirms that frailty is a complex and worrying geriatric syndrome. As the world’s population ages, frailty is becoming a serious issue for public and global health. Thus, it is crucial for frailty to be considered a holistic phenomenon with a multi-factor approach rather than merely a physical condition. This requires more research addressing multiple domains to target its prevention and reversal. Our findings indicate that reversing frailty requires that a person first be considered frail, regardless of how frailty is assessed. Although we discovered different ways of assessing frailty among the studies, a key highlight is the fact that the ability to reverse frailty may depend on how frailty is defined and measured. Hence, a consensus on what reverse frailty means is necessary. A promising but challenging area for future research could be qualitative analysis that explores frail older adults’ lived experiences and perspectives. This will guide the development and implementation of possible interventions to reverse this critical geriatric syndrome.

### Electronic supplementary material

Below is the link to the electronic supplementary material.

Supplementary Material 1

Supplementary Material 2

Supplementary Material 3

Supplementary Material 4

## Data Availability

Data supporting the findings of this study are available in the article [and its supplementary information files].

## Abbreviations

BMI: Body Mass Index
CES-D: Center for Epidemiologic Studies Depression Scale
MEDLINE: Medical Literature Analysis and Retrieval System Online
COPD: Chronic obstructive pulmonary disease
CPR: Cardiopulmonary resuscitation
Dur: Duration
FI: Frailty Index
FP: Frailty Phenotype
Freq: Frequency
GDS: Geriatric Depression Scale
HI: High intensity
iADL: Instrumental Activities of Daily Living
JBI: Joanna Briggs Institute
KCL: Kihon checklist
MEP: Multi-component Exercise Program
MeSH: Medical Subject Headings
PIFU: Post-intervention follow-up
PRESS: Peer Review of Electronic Search Strategies
PRISMA: Preferred Reporting Items for Systematic Reviews and Meta-Analyzes
RAI-HC: Resident Assessment Instrument-Home Care
RCTs: Randomized control trials
RMR: Resting metabolic rate
RT: Resistance training
SPPB: Short Physical Performance Battery
TIDieR: Template for Intervention Description and Replication
StaRI: Standards for Reporting Implementation Studies
wks: Weeks
yrs: years
m: months
