# Supplementary material for: Reversing frailty in older adults: a scoping review
Source: BMC Geriatr. 2023 Nov 17;23:751. doi: 10.1186/s12877-023-04309-y (PMC10655301; doi:10.1186/s12877-023-04309-y)
Supplement: Supplementary file 1 — Supplementary Material 1 [file 12877_2023_4309_MOESM1_ESM.pdf]

PRISMA-ScR Checklist of Study

SECTION	ITEM	PRISMA-ScR CHECKLIST ITEM	REPORTED ON PAGE #
TITLE			
Title	1	Reversing frailty in older adults: A scoping Review	
ABSTRACT			
Structured summary	2	Abstract presented in the manuscript section	
INTRODUCTION			
Rationale	3	In this study, the review process is explained in detail, with a conceptual framework for frailty identified, along with examples on the types of interventions implemented. These led to identifying the research questions, and the reason why a scoping review approach is credible (i.e., lack of a previous comprehensive review done in this area to map the evidence around interventions that achieve frailty reversal as an outcome)	Click here to enter text.
Objectives	4	The Questions being addressed are:<br>What is the available literature on interventions that achieve frailty reversal as an outcome?<br>What does it mean to reverse frailty?<br>They follow the PCC format of P-Population (older adults over 65yrs who are frail), C-Concept (reversing frailty), and C-Context (all contexts).	Click here to enter text.
METHODS			
Protocol and registration	5	Protocol developed, not registered	Not applicable
Eligibility criteria	6	Eligibility criteria clearly stated, along with its rationale	Click here to enter text.
Information sources*	7	Databases and grey literature sources used for the literature search identified but dates not included.	Click here to enter text.

SECTION	ITEM	PRISMA-ScR CHECKLIST ITEM	REPORTED ON PAGE #
		These will be done for the actual study.	
Search	8	A full electronic search strategy for ovid-MEDLINE is provided, and it was developed and refined with the help of a librarian.	
Selection of sources of evidence†	9	The studies for the review were selected based on the inclusion criteria. Covidence software was used to facilitate the process	
Data charting process‡	10	We charted the data using google Forms and Microsoft Excel.	Click here to enter text.
Data items	11	Data items were sought based on the research question, need for quality appraisal, tracking missing data, and using the TIDIER checklist for reporting interventions.	
Critical appraisal of individual sources of evidence§	12	Appraisal done using the JBI checklist for critical appraisal	
Synthesis of results	13	Using a Descriptive and narrative approach	
RESULTS			
Selection of sources of evidence	14	Section done	
Characteristics of sources of evidence	15	Section done	
Critical appraisal within sources of evidence	16	Section done	
Results of individual sources of evidence	17	Section done	
Synthesis of results	18		
DISCUSSION			

SECTION	ITEM	PRISMA-ScR CHECKLIST ITEM	REPORTED ON PAGE #
Summary of evidence	19	Section done	
Limitations	20	Limitation of study due to limited number of databases used acknowledged.	
Conclusions	21	Section done	
FUNDING			
Funding	22	Not Applicable	Not applicable

JBI = Joanna Briggs Institute; PRISMA-ScR = Preferred Reporting Items for Systematic reviews and Meta-Analyzes extension for Scoping Reviews (Tricco *et al.,* 2018)
