# Supplementary material for: Reversing frailty in older adults: a scoping review
Source: BMC Geriatr. 2023 Nov 17;23:751. doi: 10.1186/s12877-023-04309-y (PMC10655301; doi:10.1186/s12877-023-04309-y)
Supplement: Supplementary file 2 — Supplementary Material 2 [file 12877_2023_4309_MOESM2_ESM.pdf]

Articles Excluded with Reasons (n=251)

Duplicate Study (n=15)

Title	Authors	Year	Journal	Volume	Issue	Pages
A systematic review of exercise programs for the frail elderly.	Paw MJM; van Uffelen JGZ; Riphagen II; van Mechelen W	2007	Long-Term Care Interface	8	6	14-19
Singapore frailty intervention trial: Effect of frailty reversal on reducing depressive symptoms	Nyunt, Ma Shwe Zin; Feng, Liang; Feng, Lei; Ng, Tze Pin; Niti, Mathew; Tan, Boon Yeow; Chan, Gribson; Chan, Sue Mei; Ann, Khoo Sue; Yap, Philip; Yap, Keng Bee	2014	Annals of the Academy of Medicine Singapore	43	10 SUPPL. 1	S29-S30
Study to assess the effects of a nutritional formula plus exercise intervention in the functional capacities of frail institutionalized elderly patients: ACTIVNES study	Abizanda, P.; Diez, M.; Perez, V.; Da Silva, A.; Estrella, J.; Araujo, K.; Barcons, N.; Tuset, A.	2014	European Geriatric Medicine	5	SUPPL. 1	S78
Findings in Clinical Trials and Studies Reported from University Hospital (A Multicomponent Exercise Intervention that Reverses Frailty and Improves Cognition, Emotion, and Social Networking in the Community-Dwelling Frail Elderly: A Randomized ...).(Clin	Francisco José Tarazona-Santabalbina , Mari Carmen Gómez-Cabrera , Pilar Pérez-Ros , Francisco Miguel Martínez-Arnau , Helena Cabo , Konstantina Tsaparas , Andrea Salvador-	2016	Clinical Trials Week	15	5	426-33

	Pascual , Leocadio Rodriguez-Mañas , José Viña					
Intervention for falls: Reduced exercise and vitamin D supplementation among the institutionalized frail elderly	Higuchi, Y.; Todo, E.; Hirasima, K.; Kitagawa, T.; Ueda, T.; Ando, S.; Yasuoka, M.; Mizuno, T.; Imaoka, M.; Nakamura, K.; Kurosaki, K.; Ikeuchi, M.; Shichikawa, D.; Masue, A.	2015	Physiotherapy (United Kingdom)	101	SUPPL. 1	eS641
Effects of Exercise and Milk Fat Globule Membrane (MFGM) Supplementation on Body Composition, Physical Function, and Hematological Parameters in Community-Dwelling Frail Japanese Women: A Randomized Double Blind, Placebo-Controlled, Follow-Up Trial	Kim, H; Suzuki, T; Kim, M; Kojima, N; Ota, N; Shimotoyodome, A; Hase, T; Hosoi, E; Yoshida, H	2015	PLOS ONE	10	2	e0116256
Physical exercise or micronutrient supplementation for the wellbeing of the frail elderly? A randomised controlled trial.	Chin A Paw MJM; de Jong N; Schouten EG; van Staveren WA; Kok FJ; Chin A Paw, M J M; de Jong, N; Schouten, E G; van Staveren, W A; Kok, F J	2002	British Journal of Sports Medicine	36	2	126-131
A PHYSICAL ACTIVITY INTERVENTION TO TREAT THE FRAILTY SYNDROME - RESULTS	Cesari, M; Vellas, B; Doss, H; Gill, TM; Newman, AB; King,	2013	GERONTOLOGIST	70	2	216-22

FROM THE LIFESTYLE INTERVENTIONS AND INDEPENDENCE FOR ELDERS-PILOT (LIFE-P) STUDY	AC; Church, T; Pahor, M					
Systematic review of non-pharmacological interventions to treat well-defined sarcopenia and physical frailty	Lozano-Montoya, I.; Correa-Perez, A.; Abraha, I.; Cherubini, A.; Soiza, R.L.; O'Mahony, D.	2017	European Geriatric Medicine	8	Supplement 1	S112
Individualized home-based exercise and nutrition interventions improve frailty in older adults: a randomized controlled trial (vol 16, 119, 2019)	Hsieh, TJ; Su, SC; Chen, CW; Kang, YW; Hu, MH; Hsu, LL; Wu, SY; Chen, L; Chang, HY; Chuang, SY; Pan, WH; Hsu, CC	2019	INTERNATIONAL JOURNAL OF BEHAVIORAL NUTRITION AND PHYSICAL ACTIVITY	16	1	119
Effects of nutritional intervention and physical training on energy intake, resting metabolic rate and body composition in frail elderly. A randomised, controlled pilot study	Lammes, E.; Rydwik, E.; Akner, Gunnar	2012	Journal of Nutrition, Health and Aging	16	2	162-7
Is it possible to reverse frailty in patients with chronic obstructive pulmonary disease?	Wang, Z; Hu, XJ; Dai, QX	2020	CLINICS	75		e177
New Findings on Systolic Heart Failure from Columbia University Summarized (Can a Left Ventricular Assist Device in Individuals with Advanced Systolic Heart Failure Improve or Reverse Frailty?)	Mathew S. Maurer, , Evelyn Horn, Alex Reyentovich, Victoria Vaughan Dickson, Sean Pinney, Deena Goldwater, Nathan E. Goldstein, Omar	2017	Medical Devices & Surgical Technology Week	65	11	2383–2390.

	Jimenez, Sergio Teruya, Jeff Goldsmith, Stephen Helmke, MPH, RCDS, Melana Yuzefpolskaya,, and Gordon R Reeves					
â€œI Donâ€™t Feel Like Myselfâ€�: Treating Frailty in the Elderly With Diet	Firnhaber, Gina C; Kolasa, Kathryn M	2016	Nutrition today (Annapolis)	51	6	281-289
Exercise as an intervention to reverse frailty: A randomized clinical trial	Gomez-Cabrera, Mari Carmen; Tarazona-Santabalbina, Francisco Jose; Cabo, Helena; Salvador-Pascual, Andrea; Escriva, Consuelo; Rodriguez-MaÃ±as, Leocadio; ViÃ±a, Jose	2016	Free radical biology & medicine	96		S37-S37

Full Text not Available (n=27)

Title	Authors	Year	Journal	Volume	Issue	Pages
Sensible aging: using nutrient-dense foods and physical exercise with the frail elderly.	De Jong N	2001	Nutrition Today	36	4	202-207

Exercise Prescription Intervention Plan for Pre-frail and Frail Elderly in New Taipei City	Lin, HP; Tseng, YT; Lin, CH	2018	MEDICINE AND SCIENCE IN SPORTS AND EXERCISE	50	5	241-241
Acceptability of a community-based exercise and nutritional intervention in frail older adults	Jeyaseelan, Arveen; O'Donoghue, Patrick; Bambrick, Padraig; Mulcahy, Riona; Pope, George; O'Regan, Niamh; Cooke, John; Byrne, Thomas; Harrison, Michael	2020	European Geriatric Medicine	11	SUPPL 1	S220
Exercise as an intervention to reverse frailty: A randomized clinical trial	Gomez-Cabrera, Mari Carmen; Tarazona-Santabalbina, Francisco Jose; Cabo, Helena; Salvador-Pascual, Andrea; Escriva, Consuelo; Rodriguez-MaÃ±as, Leocadio; ViÃ±a, Jose	2016	Free radical biology & medicine	96		S37
Personalized multicomponent physical exercise program for the prevention and reversal of frailty in the elderly	Millan, F; Carretero, A; Garcia-Dominguez, E; Garcia-Correas, A; Garcia-Dominguez, C; De la Rosa, A; Arc-Chagnaud, C; Olaso-Gonzalez, G; Gomez-Cabrer, M; Vina, J	2021	FREE RADICAL BIOLOGY AND MEDICINE	165		

Transitional states in frailty: Implications for end of life support in heart failure	Lee, Jenny S. W.	2019	Heart Asia	11	Supplement 1	A5
Impact of nutritional supplementation and nordic walking in frail older patients in geriatrics department of tertiary care hospital in India	Kandel, R.; Chatterjee, P.; Kumar, V.; Gopalan, V.; Ambashtha, A.; Jathar, S.; Dey, A.B.	2014	European Geriatric Medicine	5	SUPPL. 1	S127-S128
Reversibility of frailty in LVAD and heart transplant patients	Macdonald, Peter S.	2019	Heart Asia	11	Supplement 1	A4-A5
Correction to: Individualized home-based exercise and nutrition interventions improve frailty in older adults: a randomized controlled trial...Hsieh TJ, et al. Individualized home-based exercise and nutrition interventions improve frailty in older adults:	Hsieh, Tsung-Jen; Su, Shin-Chang; Chen, Chun-Wei; Kang, Yaw-Wen; Hu, Ming-Hsia; Hsu, Li-Lin; Wu, Szu-Yun; Chen, Likwang; Chang, Hsing-Yi; Chuang, Shao-Yuan; Pan, Wen-Harn; Hsu, Chih-Cheng	2019	International Journal of Behavioral Nutrition & Physical Activity	16	1	136
The ex-frail CKD trial: A pilot randomised controlled trial of a home-based exercise programme for pre-frail and frail, older adults with chronic kidney disease	Brady, Mark; Dhaygude, Ajay; Nixon, Andrew; Bampouras, Theodoros; Gooch, Helen; Young, Hannah; Finlayson, Kenneth; Pendleton, Neil; Mitra, Sandip	2020	Nephrology Dialysis Transplantation	35	SUPPL 3	P095

Can an LVAD reverse the frailty phenotype in advanced heart failure?	Jimenez, O.G.; Teruya, S.; Alvarez, J.; Maurer, M.; Horn, E.; Pinney, S.; Goldwater, D.; Reyntovich, A.; Dickson, V.; Gordon, R.	2016	Journal of the American Geriatrics Society	64	SUPPL. 1	S216
The effect of task-oriented activities in the pre-frail older people	Savvakis, Ioannis; Stratidaki, Eirini; Aravantinou-Karlatou, Antonia; Patelarou, Athina; Kleisiaris, Christos; Adamakidou, Theodoula; Panagiotakis, Simeon	2020	European Geriatric Medicine	11	SUPPL 1	S217
Short-and long-term effects of a tailored multicomponent exercise programme on functional capacity in older adults living in nursing homes: pilot results from the heal study	Courel-Ibanez, J.; Munoz-Gomez, M.D.M.; Garcia Conesa, S.; Buendia Romero, A.; Gomez Vazquez, S.	2020	Osteoporosis International	31	SUPPL 1	S602
A multifactorial interdisciplinary intervention reduces frailty, increases function and is cost-effective in older adults who are frail: Randomised controlled trial	Cameron, I.D.; Fairhall, N.; Sherrington, C.; Lord, S.; Susan, K.	2015	Physiotherapy (United Kingdom)	101	SUPPL. 1	eS371-eS372
Does a multifactorial interventional programme on falls prevention in older fallers improve frailty	Tan, P.J.; Khor, H.M.; Saedon, N.I.; Kamaruzzaman, S.B.; Tan, M.P.	2017	Age and Ageing	46	Supplement 2	

outcomes? Preliminary results from the Malaysian falls assessment intervention trial (MYFAIT)						
DEFRAIL (diet and exercise for frailty): The effect of a novel multi-component group exercise program and protein supplementation on frailty in older adults	Bambrick, Padraig; Mulcahy, Riona; Cooke, John; Byrne, Thomas; Harrison, Michael; Phelan, Niamh; Grant, Emma	2020	European Geriatric Medicine	11	SUPPL 1	S223
Frailty and clinical outcomes of transcatheter aortic valve replacement	Cheema, A.	2018	Cardiology (Switzerland)	140	Supplement 1	175
Promoting active ageing through a physical exercise program aimed at reducing frailty and risk of falling among older adults.	Alhambra-BorrÃ¡s, Tamara; Valia-Cotanda, Elisa; Dura-Ferrandis, Estrella; GarcÃ©s-Ferrer, Jordi; Quel-TejÃ³n, BelÃ©n	2017	International Journal of Integrated Care (IJIC)	17	5	1-8
[Intervention effects of inclusive support in an "exercise and a nutritional community-based prevention program" for pre-frail elderly individuals].	Fukasaku, Takako; Okuno, Junko; Tomura, Shigeo; Seino, Satoshi; Kim, Mi-Ji; Yabushita, Noriko; Okura, Tomohiro; Tanaka, Kiyoji; Yanagi, Hisako	2011	[Nihon koshu eisei zasshi] Japanese journal of public health	58	6	420-32
Using a group exercise program to improve the flexibility of frail older adults	Lazowski, DA; Ecclestone, NA; Paterson, DH;	1997	JOURNAL OF AGING AND PHYSICAL ACTIVITY	5	4	376-376

living in long-term care institutions	Fitzgerald, C; Jones, G; TudorLocke, CE					
Meta-analysis of Primary Care Interventions to Address Frailty Among Adults Aged 65+...67th Annual & Scientific Meeting of the Irish Gerontological Society, Innovation, Advances and Excellence in Ageing, September 26-28, 2019, Cork, Ireland	Macdonald, Steve; Travers, John; Eidí n Ní Shé ; Bailey, Jade; Romero-Ortuno, Roman; Keyes, Michael; O'Shea, Diarmuid; Cooney, Marie Therese	2019	Age & Ageing	48		iii17-iii65
Training and de-training effects: One year follow-up of a 3-month resistance exercise program in the pre-frail elderly	Wu, H.-H.; Tseng, T.-J.; Gi, B.-H.; Lin, T.-Y.; Lin, P.-S.; Liao, T.-H.	2015	Physiotherapy (United Kingdom)	101	SUPPL. 1	eS882
Effect of Exercise Intervention on Frailty, Muscular Function, and Self-Efficacy in Daily Activities of Life for the Elder Population	Chen, YL; Huang, CH; Chang, YS	2016	JOURNAL OF AGING AND PHYSICAL ACTIVITY	24		S87-S87
EFFECT OF DIETARY AND EXERCISE INTERVENTIONS IN SARCOPENIC, PRE-FRAIL AND FRAIL OLDER ADULTS	Hida, A; Anton, S; Mankowski, R; Layne, A; Solberg, L; Mainous, A; Buford, T	2017	ANNALS OF NUTRITION AND METABOLISM	71		869-869
Modest functional exercise protocol with frail elders improves functional capacity: a series of case studies.	Blain A; McKnight J; Hutchinson K; Lowe S; Fitzpatrick D	2004	Journal of Geriatric Physical Therapy	27	3	113-113

Effects of an exercise protocol in frail and pre-frail elderly	Amaro, R.; Alegria, N.; Ramalhinho, M.; Afonso, G.; Morais, D.; Pereira, F.; Araujo, G.; Miguel, S.; Santos, O.	2016	European Geriatric Medicine	7	Supplement 1	S151
Nutrition, or Nutrition and Exercise? A Systematic Review of Interventions in Frail Elderly People	Saleh, RA; Lirette, S; Elisson, J; Wright, M; Cleinman, A	2015	JOURNAL OF THE AMERICAN GERIATRICS SOCIETY	63		S61-S61

Non-English or French (n= 10)

**Title**	**Authors**	**Year**	**Journal**	**Volume**	**Issue**	**Pages**
[Physical exercise in the frail elderly: an update].	Casas Herrero, Alvaro; Cadore, Eduardo L; Martinez Velilla, Nicolas; Izquierdo Redin, Mikel	2015	Revista espanola de geriatria y gerontologia	50	2	74-81
[A systematic review of intervention programs for frail elderly people enrolled in the Japanese social long-term care insurance system].	Ukawa, Shigekazu; Tamakoshi, Akiko; Sakamoto, Ai	2015	[Nihon koshu eisei zasshi] Japanese journal of public health	62	1	3-19
[Interventions for frailty and sarcopenia in community-dwelling elderly women].	Kim, Hunkyung	2012	Nihon Ronen Igakkai zasshi. Japanese journal of geriatrics	49	6	726-30

Effect of strength exercise with elastic bands and aerobic exercise in the treatment of frailty of the elderly patient with type 2 diabetes mellitus.	Garcia Diaz, Eduardo; Alonso Ramirez, Javier; Herrera Fernandez, Nuria; Peinado Gallego, Concha; Perez Hernandez, Domingo de Guzman	2019	Endocrinologia, diabetes y nutricion	66	9	563-570
[Effects of an intervention program for community-dwelling elderly to improve frailty and dietary habits].	Kawabata, Teruko; Takemi, Yukari; Murayama, Hiroshi; Nishi, Mariko; Shimizu, Yumiko; Narita, Miki; Kim, Mi-Ji; Shinkai, Shoji	2015	[Nihon koshu eisei zasshi] Japanese journal of public health	62	4	169-81
Nutritional, Physical, Cognitive, and Combination Interventions and Frailty Reversal among Older Adults: A Randomized Controlled Trial	Braun, T	2016	PHYSIOSCIENCE	12	4	165-U61
[Effectiveness of physical exercise on fitness in frail older adults: A systematic review of randomised trials].	Viladrosa, Maria; Casanova, Carles; Ghiorghies, Angela Claudia; Jurschik, Pilar	2017	Revista espanola de geriatria y gerontologia	52	6	332-341
Physical exercise as an efficient intervention in frail elderly persons	Herrero, AC; Izquierdo, M	2012	ANALES DEL SISTEMA SANITARIO DE NAVARRA	35	1	69-85
[Effects of a multifactorial intervention for improving frailty on risk of long-term care insurance certification, death, and long-term care cost among community-dwelling older adults: A quasi-	Yokoyama, Yuri; Seino, Satoshi; Mitsutake, Seigo; Nishi, Mariko; Murayama, Hiroshi; Narita, Miki; Ishizaki, Tatsuro; Nofuji, Yu;	2020	[Nihon koshu eisei zasshi] Japanese journal of public health	67	10	752-762

experimental study using propensity score matching].	Kitamura, Akihiko; Shinkai, Shoji					
Physical exercise as an efficient intervention in frail elderly persons physical exercise as an efficient intervention in frail elderly persons	Casas Herrero, A.; Izquierdo, M.	2012	Anales del Sistema Sanitario de Navarra	35	1	69-85

Pre and frail outcome reported combined (n= 23)

Title	Authors	Year	Journal	Volume	Issue	Pages
Home-based exercise for people living with frailty and chronic kidney disease: A mixed-methods pilot randomised controlled trial	Nixon, AC; Bampouras, TM; Gooch, HJ; Young, HML; Finlayson, KW; Pendleton, N; Mitra, S; Brady, ME; Dhaygude, AP	2021	PLOS ONE	16	7	e0251652
Integrated care for geriatric frailty and sarcopenia: a randomized control trial	Chan, DC; Tsou, HH; Chang, CB; Yang, RS; Tsauo, JY; Chen, CY; Hsiao, CF; Hsu, YT; Chen, CH; Chang, SF; Hsiung, CA; Kuo, KN	2017	JOURNAL OF CACHEXIA SARCOPENIA AND MUSCLE	8	1	78-88
Individualized home-based exercise and nutrition interventions improve frailty in older adults: a randomized controlled trial.	Hsieh, Tsung-Jen; Su, Shin-Chang; Chen, Chun-Wei; Kang, Yaw-Wen; Hu, Ming-Hsia; Hsu, Li-Lin; Wu, Szu-Yun; Chen, Likwang; Chang, Hsing-Yi; Chuang, Shao-Yuan; Pan, Wen-Harn; Hsu, Chih-Cheng	2019	International Journal of Behavioral Nutrition & Physical Activity	16	1	119

A Randomized Controlled Pilot Exercise and Protein Effectiveness Supplementation Study (EXPRESS) on Reducing Frailty Risk in Community-Dwelling Older People.	Jadczak, Agathe Daria; Visvanathan, Renuka; Barnard, Robert; Luscombe-Marsh, Natalie	2021	Journal of nutrition in gerontology and geriatrics	40	1	26-45
Chair-based exercise programs in institutionalized older women: Salivary steroid hormones, disabilities and frailty changes	Furtado, GE; Carvalho, HM; Loureiro, M; Patricio, M; Uba-Chupel, M; Colado, JC; Hogervorst, E; Ferreira, JP; Teixeira, AM	2020	EXPERIMENTAL GERONTOLOGY	130		110790
Effect of various exercises on frailty among older adults with subjective cognitive concerns: a randomised controlled trial.	Huang, Chi Hsien; Umegaki, Hiroyuki; Makino, Taeko; Uemura, Kazuki; Hayashi, Takahiro; Kitada, Tomoharu; Inoue, Aiko; Shimada, Hiroyuki; Kuzuya, Masafumi	2020	Age & Ageing	49	6	1011-1019
How to Improve the Functional Capacity of Frail and Pre-Frail Elderly People? Health, Nutritional Status and Exercise Intervention. The EXERNET-Elder 3.0 Project	Fernandez-Garcia, AI; Gomez-Cabello, A; Moradell, A; Navarrete-Villanueva, D; Perez-Gomez, J; Ara, I; Pedrero-Chamizo, R; Subias-Perie, J; Muniz-Pardos, B; Casajus, JA; Vicente-Rodriguez, G	2020	SUSTAINABILITY	12	15	6246
Cognitive Effects of Multi-Domain Interventions Among Pre-Frail and Frail	Tze Pin Ng; Ling Hui Audrey Ling; Liang Feng; Ma Shwe Zin Nyunt; Lei Feng; Niti, Mathew; Boon Yeow	2018	Journals of Gerontology Series A:	73	6	806-812

Community-Living Older Persons: Randomized Controlled Trial.	Tan; Chan, Gribson; Khoo, Sue Anne; Sue Mei Chan; Yap, Philip; Keng Bee Yap; Ng, Tze Pin; Ling, Ling Hui Audrey; Feng, Liang; Nyunt, Ma Shwe Zin; Feng, Lei; Tan, Boon Yeow; Chan, Sue Mei; Yap, Keng Bee		Biological Sciences & Medical Sciences			
Effects of horticultural therapy: Perspectives of frail and pre-frail older nursing home residents	Lo, SKL; Lam, WYY; Kwan, RYC; Tse, MMY; Lau, JKH; Lai, CKY	2019	NURSING OPEN	6	3	1230-1236
Effects of dehydroepiandrosterone (DHEA) on cardiovascular risk factors in older women with frailty characteristics	Kleppinger, A.; Burleson, J.A.; Kenny, A.M.; Feinn, R.; Brindisi, J.; Boxer, R.S.	2010	Age and Ageing	39	4	451-458
The effect of the group-based Otago exercise program on frailty among nursing home older adults with cognitive impairment.	Feng, Hong; Zou, Zhijie; Zhang, Qing; Wang, Liang; Ouyang, Yan-Qiong; Chen, Zhongwan; Ni, Zhao	2021	Geriatric Nursing	42	2	479-483
Effectiveness of combined exercise and nutrition interventions in prefrail or frail older hospitalised patients: a systematic review and meta-analysis	Han, CY; Miller, M; Yaxley, A; Baldwin, C; Woodman, R; Sharma, Y	2020	BMJ OPEN	10	12	e040146

Adherence Is More Than Just Being Present: Example of a Lay-Led Home-Based Programme with Physical Exercise, Nutritional Improvement and Social Support, in Prefrail and Frail Community-Dwelling Older Adults	Lackinger, C; Grabovac, I; Haider, S; Kapan, A; Winzer, E; Stein, KV; Dorner, TE	2021	INTERNATIONAL JOURNAL OF ENVIRONMENTAL RESEARCH AND PUBLIC HEALTH	18	8	4192
Effects of a Multicomponent Exercise Program, a Detraining Period and Dietary Intake Prediction of Body Composition of Frail and Pre-Frail Older Adults from the EXERNET Elder 3.0 Study	Moradell, A; Navarrete-Villanueva, D; Fernandez-Garcia, AI; Sagarra-Romero, L; Marin-Puyalto, J; Perez-Gomez, J; Gesteiro, E; Ara, I; Casajus, JA; Gomez-Cabello, A; Rodriguez, GV	2020	SUSTAINABILITY	12	23	9894
Randomized Comparison of Exercise Intervention Versus Usual Care in Older Adult Patients with Frailty After Acute Myocardial Infarction.	Sanchis, Juan; Sastre, Clara; Ruescas, Arantxa; Ruiz, Vicente; Valero, Ernesto; Bonanad, Clara; GarcÃa-Blas, Sergio; FernÃ¡ndez-Cisnal, AgustÃn; GonzÃ¡lez, Jessika; MiÃ±ana, Gema; NÃºÃ±ez, Julio	2021	American Journal of Medicine	134	3	383-383
Effects of a Primary Care-Based Multifactorial Intervention on Physical and Cognitive Function in Frail, Elderly Individuals:	Romera-Liebana, Laura; Orfila, Francesc; Segura, Josep Maria; Real, Jordi; Fabra, Maria LluÃ¯sa; MÃ¶ller, Mercedes; Lancho, Santiago; Ramirez, Anna; Marti, Nuria; Cullell, Montserrat; Bastida, Nuria; Martinez, Dolors; GinÃ©,	2018	Journals of Gerontology Series A: Biological Sciences & Medical Sciences	73	12	1688-1674

A Randomized Controlled Trial.	Maria; CendrÃ³s, Patricia; Bistuer, Anna; Perez, Elena; Fabregat, Maria Assumpta; Foz, GonÃ§al					
Effects of exercise training on frailty in community-dwelling older adults: results of a randomized, controlled trial.	Binder EF; Schechtman KB; Ehsani AA; Steger-May K; Brown M; Sinacore DR; Yarasheski KE; Holloszy JO	2002	Journal of the American Geriatrics Society	50	12	1921-1928
Impact of a lay-led home-based intervention programme on quality of life in community-dwelling pre-frail and frail older adults: a randomized controlled trial.	Kapan, A.; Winzer, E.; Haider, S.; Titze, S.; Schindler, K.; Lackinger, C.; Dorner, T. E.	2017	BMC Geriatrics	17	1	154
Effects and feasibility of exercise therapy combined with branched-chain amino acid supplementation on muscle strengthening in frail and pre-frail elderly people requiring long-term care: a crossover trial.	Ikeda, Takashi; Aizawa, Junya; Nagasawa, Hiroshi; Gomi, Ikuko; Kugota, Hiroyuki; Nanjo, Keigo; Jinno, Tetsuya; Masuda, Tadashi; Morita, Sadao	2016	Applied Physiology, Nutrition & Metabolism	41	4	438-445
Protein Supplementation Improves Physical Performance in Frail Elderly People: A	Tieland, Michael; van de Rest, Ondine; Dirks, Marlou L.; van der Zwaluw, Nikita; Mensink, Marco;	2012	Journal of the American Medical Directors Association	13	8	720-726

Randomized, Double-Blind, Placebo-Controlled Trial.	van Loon, Luc J.C.; de Groot, Lisette C.P.G.M.					
Efficacy of multidomain interventions to improve physical frailty, depression and cognition: data from cluster-randomized controlled trials	Taiwan Hlth Promotion Intervention; Chen, LK; Hwang, AC; Lee, WJ; Peng, LN; Lin, MH; Neil, DL; Shih, SF; Loh, CH; Chiou, ST	2020	JOURNAL OF CACHEXIA SARCOPENIA AND MUSCLE	11	3	650-662
Effects of Resistance Exercise Training on Cognitive Function and Physical Performance in Cognitive Frailty: A Randomized Controlled Trial.	Yoon, D. H.; Lee, Jun-Young; Song, Wook	2018	Journal of Nutrition, Health & Aging	22	8	944-951
A realist review to understand the efficacy and outcomes of interventions designed to minimise, reverse or prevent the progression of frailty.	Gwyther, Holly; Bobrowicz-Campos, Elzbieta; Luis Alves ApÃ³stolo, JoÃ£o; Marcucci, Maura; Cano, Antonio; Holland, Carol	2018	Health Psychology Review	12	4	382-404

Wrong patient population <65yrs/frail state not mentioned (n= 87)

Title	Authors	Year	Journal	Volume	Issue	Pages

THE EFFECT OF PHYSICAL EXERCISE ON FRAIL OLDER PERSONS: A SYSTEMATIC REVIEW	Silva, RB; Aldoradin-Cabeza, H; Eslick, GD; Phu, S; Duque, G	2017	JOURNAL OF FRAILTY & AGING	6	2	91-96
Reversibility of Frailty after Lung Transplantation	Montgomery, E; Macdonald, PS; Newton, PJ; Chang, S; Wilhelm, K; Jha, SR; Malouf, M	2020	JOURNAL OF TRANSPLANTATION	37	4	S249
Dehydroepiandrosterone combined with exercise improves muscle strength and physical function in frail older women.	Kenny AM; Boxer RS; Kleppinger A; Brindisi J; Feinn R; Burleson JA	2010	Journal of the American Geriatrics Society	58	9	1707-1714
Possible Sarcopenia and Impact of Dual-Task Exercise on Gait Speed, Handgrip Strength, Falls, and Perceived Health	Merchant, RA; Chan, YH; Hui, RJY; Lim, JY; Kwek, SC; Seetharaman, SK; Au, LSY; Morley, JE	2021	FRONTIERS IN MEDICINE	8		
Nutrition and Frailty: Opportunities for Prevention and Treatment.	Ni Lochlainn, Mary; Cox, Natalie J.; Wilson, Thomas; Hayhoe, Richard P. G.; Ramsay, Sheena E.; Granic, Antoneta; Isanejad, Masoud; Roberts, Helen C.; Wilson, Daisy; Welch, Carly; Hurst, Christopher; Atkins, Janice L.; MendonÃ§a, Nuno; Horner, Katy; Tuttiett, Esme R.; Morgan, Yvie; Heslop, Phil; Williams,	2021	Nutrients	13	7	2349-2349

	Elizabeth A.; Steves, Claire J.; Greig, Carolyn					
Exercise and/or Dietary Varieties and Incidence of Frailty in Community-Dwelling Older Women: A 2-Year Cohort Study.	Osuka, Yosuke; Kojima, N.; Yoshida, Y.; Kim, M.; Won, CW.; Suzuki, T.; Kim, H.	2019	Journal of Nutrition, Health & Aging	23	5	425-430
Effects of passive exercise training on physical and psychological variables of elderly participants living in long-term care: a cross sectional study.	Brenner, Ingrid	2009	Perspectives (Gerontological Nursing Association (Canada))	33	4	7-14
Obesity and Physical Frailty in Older Adults: A Scoping Review of Lifestyle Intervention Trials.	Porter Starr, Kathryn N.; McDonald, Shelley R.; Bales, Connie W.	2014	Journal of the American Medical Directors Association	15	4	240-250
Effects of lower limb resistance exercise on muscle strength, physical fitness, and metabolism in pre-frail elderly patients: a randomized controlled trial.	Lai, Xiaoxing; Bo, Lin; Zhu, Hongwei; Chen, Baoyu; Wu, Zhao; Du, Hongdi; Huo, Xiaopeng	2021	BMC geriatrics	21	1	447
A multicomponent frailty intervention for socioeconomically vulnerable older adults: a designed-delay study	Jang, IY; Jung, HW; Park, H; Lee, CK; Yu, SS; Lee, YS; Lee, E; Glynn, RJ; Kim, DH	2018	CLINICAL INTERVENTIONS IN AGING	13		1799-1814

Tai Chi is an effective form of exercise to reduce markers of frailty in older age	Kasim, NF; van Zanten, JV; Aldred, S	2020	EXPERIMENTAL GERONTOLOGY	135		110925
Involving older people in co-designing an intervention to reverse frailty and build resilience.	Travers, John; Romero-Ortuno, Roman; Ni She, Eidin; Cooney, Marie-Therese	2021	Family practice	39	1	200-206
Frailty and exercise interventions Evidence and barriers for exercise programs	Freiberger, E; Kemmler, W; Siegrist, M; Sieber, C	2016	ZEITSCHRIFT FUR GERONTOLOGIE UND GERIATRIE	49	7	606-611
Feasibility, safety, acceptability, and functional outcomes of playing Nintendo Wii Fit PlusTM for frail older adults: A randomized feasibility clinical trial.	Gomes, Gisele Cristine Vieira; SimÃµes, Maria do Socorro; Lin, Sumika Mori; Bacha, JÃ©ssica Maria Ribeiro; Viveiro, Larissa Alamino Pereira; Varise, Eliana Maria; Carvas Junior, Nelson; Lange, Belinda; Jacob Filho, Wilson; Pompeu, JosÃ© Eduardo	2018	Maturitas	118		20-28
Nonpharmacological interventions to treat physical frailty and sarcopenia in older patients: a systematic overview - the SENATOR Project ONTOP Series	Lozano-Montoya, I; Correa-Perez, A; Abraha, I; Soiza, RL; Cherubini, A; O'Mahony, D; Cruz-Jentoft, AJ	2017	CLINICAL INTERVENTIONS IN AGING	12		721-740

Effects of exercise programs on falls and mobility in frail and pre-frail older adults: a multicenter randomized controlled trial.	Faber MJ; Bosscher RJ; Paw MJC; van Wieringen PC	2006	Archives of Physical Medicine & Rehabilitation	87	7	885-896
A Multicomponent Intervention Program to Improve Physical Function and Frailty in Vulnerable Older Adults: A Designed-Delay Intervention Study	Jang, I; Jung, H; Lee, Y; Lee, E; Kim, D	2018	JOURNAL OF THE AMERICAN GERIATRICS SOCIETY	13		1799–1814
LIFE-SUSTAINING INTERVENTIONS IN FRAIL ELDERLY PERSONS - TALKING ABOUT CHOICES	KELLOGG, FR; CRAIN, M; CORWIN, J; BRICKNER, PW	1992	ARCHIVES OF INTERNAL MEDICINE	152	11	2317-2320
The effects of exercise on strength and physical performance in frail older people: A systematic review	Katharine CM Nash	2012	Reviews in Clinical Gerontology	22	4	274-285
A primary care approach to frailty and sarcopenia	Woo, Jean	2019	Aging Medicine and Healthcare	10	Supplement 1	2
Does Home-Based Exercise Improve the Physical Function of Prefrail Older Women?	Garcia, RNSD; Costa, SN; Garcia, EDSD; Bento, PCB	2021	REJUVENATION RESEARCH	24	1	13-Jun
Interventions to prevent, delay or reverse frailty in older people: a journey	Marcucci, Maura; Damanti, Sarah; Germini, Federico; Apostolo, Joao;	2019	BMC Medicine	17	1	193-193

towards clinical guidelines.	Bobrowicz-Campos, Elzbieta; Gwyther, Holly; Holland, Carol; Kurpas, Donata; Bujnowska-Fedak, Maria; Szwamel, Katarzyna; Santana, Silvina; Nobili, Alessandro; D'Avanzo, Barbara; Cano, Antonio					
Interventions to improve the outcomes of frail people having surgery: A systematic review	McIsaac, DI; Jen, T; Mookerji, N; Patel, A; Lalu, MM	2017	PLOS ONE	12	12	e0190071
A multicomponent exercise intervention to improve physical functioning, cognition and psychosocial well-being in elderly nursing home residents: a study protocol of a randomized controlled trial in the PROCARE (prevention and occupational health in long-t	Cordes, Thomas; Bischoff, Laura L.; Schoene, Daniel; Schott, Nadja; Voelcker-Rehage, Claudia; Meixner, Charlotte; Appelles, Luisa-Marie; Bebenek, Michael; Berwinkel, Andre; Hildebrand, Claudia; JÃ¶llenbeck, Thomas; Johnen, Bettina; Kemmler, Wolfgang; Klotzbier, Thomas; Korbus, Heide; Rudisch, Julian; Vogt, Lutz; Weigelt, Matthias; Wittelsberger, Rita; Zwingmann, Katharina	2019	BMC Geriatrics	19	1	369

Cost analysis of a community-based exercise and nutritional intervention in frail older adults	Walsh, Joseph; Bambrick, Padraig; Pope, George; O'Regan, Niamh; Harrison, Michael; Byrne, Thomas; Mulcahy, Riona; Cooke, John	2020	European Geriatric Medicine	11	SUPPL 1	S223
Feasibility of a community-based Functional Power Training program for older adults	Tan, QLL; Chye, LMY; Ng, DHM; Chong, MS; Ng, TP; Wee, SL	2018	CLINICAL INTERVENTIONS IN AGING	13		309-316
Effect of an integrated care model for pre-frail and frail older people living in community.	Yu, Ruby; Tong, Cecilia; Woo, Jean	2020	Age & Ageing	49	6	1048-1055
Motoric cognitive risk syndrome, physio-cognitive decline syndrome, cognitive frailty and reversibility with dual-task exercise	Merchant, RA; Chan, YH; Hui, RJY; Tsoi, CT; Kwek, SC; Tan, WM; Lim, JY; Sandrasageran, S; Wong, BLL; Chen, MZ; Ng, SE; Morley, JE	2021	EXPERIMENTAL GERONTOLOGY	150		111362
A case of chronic dizziness and frailty that improved with a combination of exercise and medical guidance	Kawamura, Koki; Kondo, Izumi; Nakada, Takafumi; Sugiura, Saiko; Uchida, Yasue	2021	Equilibrium Research	80	2	104-111
MANAGEMENT OF FRAILTY AT INDIVIDUAL LEVEL: NARRATIVE REVIEW OF PHYSICAL ACTIVITY FROM THE	Strojnik, V; Gabrovec, B	2019	ZDRAVSTVENO VARSTVO	58	2	84-90

EUROPEAN PERSPECTIVE OF JOINT ACTION ON FRAILTY - JA ADVANTAGE						
The effects of exercise on strength and physical performance in frail older people: a systematic review.	Nash, Katharine CM	2012	Reviews in Clinical Gerontology	22	4	274-285
Randomized controlled trial to evaluate effectiveness of exercise therapy (Takizawa Program) for frail elderly.	Makita, Mitsuyo; Nakadaira, Hiroto; Yamamoto, Masaharu	2006	Environmental health and preventive medicine	11	5	221-7
Delaying and reversing frailty: a systematic review of primary care interventions	Travers, J; Romero-Ortuno, R; Bailey, J; Cooney, MT	2019	BRITISH JOURNAL OF GENERAL PRACTICE	69	678	E61-E69
Reversal of age-associated frailty by controlled physical exercise: The pre-clinical and clinical evidences	Millan, F.; Salvador-Pascual, A.; Correas, A.G.; Olaso-Gonzalez, G.; De la Rosa, A.; Carretero, A.; Gomez-Cabrera, M.C.; Vina, J.; Arc-Chagnaud, C.	2019	Sports Medicine and Health Science	1	1	33-39
The Atlanta FICSIT study: two exercise interventions to reduce frailty in elders.	Wolf SL; Kutner NG; Green RC; McNeely E	1993	Journal of the American Geriatrics Society	41	3	329-332
Social isolation and loneliness as related to progression and reversion of frailty in the Survey of	Jarach, Carlotta Micaela; Tettamanti, Mauro;	2021	Age & Ageing	50	1	258-262

Health Aging Retirement in Europe (SHARE).	Nobili, Alessandro; D'avanzo, Barbara					
The Home-based Older People's Exercise (HOPE) trial: a pilot randomised controlled trial of a home-based exercise intervention for older people with frailty.	Clegg, Andrew; Barber, Sally; Young, John; Iliffe, Steve; Forster, Anne	2014	Age & Ageing	43	5	687-695
Is It Time to Begin a Public Campaign Concerning Frailty and Pre-frailty? A Review Article	Sacha, J; Sacha, M; Sobon, J; Borysiuk, Z; Feusette, P	2017	FRONTIERS IN PHYSIOLOGY	8		484
Adherence to and effectiveness of an individually tailored home-based exercise program for frail older adults, driven by mobility monitoring: design of a prospective cohort study	Geraedts, HAE; Zijlstra, W; Zhang, W; Bulstra, S; Stevens, M	2014	BMC PUBLIC HEALTH	14		570
Reversing Frailty Levels in Primary Care Using the CARES Model.	Theou, Olga; Park, Grace H.; Garm, Antonina; Song, Xiaowei; Clarke, Barry; Rockwood, Kenneth	2017	Canadian Geriatrics Journal	20	3	105-111
Metaâ€analysis and GRADE profiles of exercise interventions for falls prevention in	Schoberer, Daniela; Breimaier, Helga E.	2020	Journal of Advanced Nursing (John Wiley & Sons, Inc.)	76	1	121-134

longâ€term care facilities.						
Mediterranean diet intervention alters the gut microbiome in older people reducing frailty and improving health status: the NU-AGE 1-year dietary intervention across five European countries	Ghosh, TS; Rampelli, S; Jeffery, IB; Santoro, A; Neto, M; Capri, M; Giampieri, E; Jennings, A; Candela, M; Turroni, S; Zoetendal, EG; Hermes, GDA; Elodie, C; Meunier, N; Brugere, CM; Pujos-Guillot, E; Berendsen, AM; De Groot, LCPGM; Feskins, EJM; Kaluza, J; Pietruszka, B; Bielak, MJ; Comte, B; Maijo-Ferre, M; Nicoletti, C; De Vos, WM; Fairweather-Tait, S; Cassidy, A; Brigidi, P; Franceschi, C; O'Toole, PW	2020	GUT	69	7	1218-1228
Frailty predicts adverse outcomes in older people with diabetes.	Ulley, Joanna; Abdelhafiz, Ahmed H.	2017	Practitioner	261	1800	17-20
Cognitive Frailty and Its Association with All-Cause Mortality Among Community-Dwelling Older Adults in Taiwan: Results from I-Lan Longitudinal Aging Study.	Liu, Li-Kuo; Chen, Chia-Hung; Lee, Wei-Ju; Wu, Yi-Hui; Hwang, An-Chun; Lin, Ming-Hsien; Shimada, Hiroyuki; Peng, Li-Ning; Loh, Ching-Hui; Arai, Hidenori; Chen, Liang-Kung	2018	Rejuvenation Research	21	6	510-517

The pulmonary rehabilitation regimen: a treatment for frailty and â€˜inflammagingâ€™?	Tanner, A; Vassallo, M; Kwan, JSK; Allen, SC	2018	British Journal of Hospital Medicine (17508460)	79	8	432-437
The role of prehabilitation in frail surgical patients: A systematic review	Milder, DA; Pillinger, NL; Kam, PCA	2018	ACTA ANAESTHESIOLOGICA SCANDINAVICA	62	10	1356-1366
Multicomponent exercise and the hallmarks of frailty: Considerations on cognitive impairment and acute hospitalization	Cadore, EL; de Asteasu, MLS; Izquierdo, M	2019	EXPERIMENTAL GERONTOLOGY	122		10-14
Review of Interventions for the Frailty Syndrome and the Role of Metformin as a Potential Pharmacologic Agent for Frailty Prevention	Espinoza, SE; Jiwani, R; Wang, J; Wang, CP	2019	CLINICAL THERAPEUTICS	41	3	376-386
A comparison of functional outcomes following a physical activity intervention for frail older adults in personal care homes.	Taylor LF; Whittington F; Hollingsworth C; Ball M; King SV; Diwan S; Rosenbloom C; Patterson V; Neel A	2003	Journal of Geriatric Physical Therapy	26	1	7-11
Physical Exercise as Therapy for Frailty	Aguirre, LE; Villareal, DT	2015	FRAILTY: PATHOPHYSIOLOGY, PHENOTYPE AND PATIENT CARE	83		83-92

The effects of exercise on the quality of life of frail older adults: A preplanned meta-analysis of the FICSIT trials	FICSIT Grp; Schechtman, KB; Ory, MG	2001	ANNALS OF BEHAVIORAL MEDICINE	23	3	186-197
EXERCISE TRAINING AND NUTRITIONAL SUPPLEMENTATION FOR PHYSICAL FRAILTY LN VERY ELDERLY PEOPLE	FIATARONE, MA; ONEILL, EF; RYAN, ND; CLEMENTS, KM; SOLARES, GR; NELSON, ME; ROBERTS, SB; KEHAYIAS, JJ; LIPSITZ, LA; EVANS, WJ	1994	NEW ENGLAND JOURNAL OF MEDICINE	330	25	1769-1775
Effects of a group-based exercise program on the mood state of frail older women after discharge from hospital.	Timonen, L; Rantanen, T; Timonen, T E; Sulkava, R	2002	International Journal of Geriatric Psychiatry	17	12	1106-1111
Health Benefits of beta-Hydroxy-beta-Methylbutyrate (HMB) Supplementation in Addition to Physical Exercise in Older Adults: A Systematic Review with Meta-Analysis	Courel-Ibanez, J; Vetrovsky, T; Dadova, K; Pallares, JG; Steffl, M	2019	NUTRIENTS	11	9	2082
Randomized Controlled Trial on the Effects of a Combined Intervention of Computerized Cognitive Training Preceded by Physical Exercise for Improving Frailty Status	Yu, R; Leung, G; Woo, J	2021	INTERNATIONAL JOURNAL OF ENVIRONMENTAL RESEARCH AND PUBLIC HEALTH	18	4	1396

and Cognitive Function in Older Adults						
THE ATLANTA FICSIT STUDY - 2 EXERCISE INTERVENTIONS TO REDUCE FRAILTY IN ELDERS	WOLF, SL; KUTNER, NG; GREEN, RC; MCNEELY, E	1993	JOURNAL OF THE AMERICAN GERIATRICS SOCIETY	41	3	329-332
Primary care interventions to address physical frailty among community-dwelling adults aged 60 years or older: A meta-analysis	Macdonald, SHF; Travers, J; She, EN; Bailey, J; Romero-Ortuno, R; Keyes, M; O'Shea, D; Cooney, MT	2020	PLOS ONE	15	2	e0228821
Exergaming as a Physical Exercise Strategy Reduces Frailty in People With Dementia: A Randomized Controlled Trial.	Karssemeijer, Esther G.A.; Bossers, Willem J.R.; Aaronson, Justine A.; Sanders, Lianne M.J.; Kessels, Roy P.C.; Olde Rikkert, Marcel G.M.	2019	Journal of the American Medical Directors Association	20	12	1502-1502
Effects of in-hospital exercise on frailty in patients with hepatocellular carcinoma	Tsuchihashi, Jin; Koya, Shunji; Hirota, Keisuke; Koga, Noboru; Narao, Hayato; Tomita, Manabu; Kawaguchi, Takumi; Nakano, Dan; Tsutsumi, Tsubasa; Torimura, Takuji; Hashida, Ryuki; Matsuse, Hiroo; Yoshio, Sachiyo; Sanada, Taku; Notsumata, Kazuo	2021	Cancers	13	2	194

Effects of Home-Based Exercise on Frailty in Patients With End-Stage Renal Disease: Systematic Review	Yoo, J; Ruppar, T; Wilbur, J; Miller, A; Westrick, JC		BIOLOGICAL RESEARCH FOR NURSING	24	1	48-63
Relationships of exercise with frailty, depression, and cognitive function in older women	Jeoung, BJ	2014	JOURNAL OF EXERCISE REHABILITATION	10	5	291-294
Association between Diet Quality and Frailty Prevalence in the Physicians' Health Study.	Ward, Rachel E.; Orkaby, Ariela R.; Chen, Jiaying; Hshieh, Tammy T.; Driver, Jane A.; Gaziano, J. Michael; Djousse, Luc	2020	Journal of the American Geriatrics Society	68	4	770-776
Effectiveness of a Community-Based Muscle Strengthening Exercise Program to Increase Muscle Strength Among Pre-frail Older Persons in Malaysia: A Pilot Study	Adnan, RNER; Din, HM; Ashari, A; Minhat, HS	2021	FRONTIERS IN PUBLIC HEALTH	9		610184
Physical frailty and its associated factors among elderly nursing home residents in China.	Liu, Weiwei; Puts, Martine; Jiang, Fen; Zhou, Chuyi; Tang, Siyuan; Chen, Sanmei	2020	BMC Geriatrics	20	1	294
Delaying and reversing frailty: A systematic review of primary care interventions	Travers, John; Cooney, Therese; Romero-Ortuno, Roman; Bailey, Jade	2018	Age and Ageing	69	678	e61-e69

BE WELL: RESULTS OF A NUTRITION, EXERCISE, AND WEIGHT MANAGEMENT INTERVENTION AMONG FRAIL OLDER ADULTS	Kogan, AC; Hart, B; Gonzalez, J; Enguidanos, S	2011	GERONTOLOGIST	32	7	889-901
Protocolized exercise improves frailty parameters and lower extremity impairment: A promising prehabilitation strategy for kidney transplant candidates	Lorenz, EC; Hickson, LJ; Weatherly, RM; Thompson, KL; Walker, HA; Rasmussen, JM; Stewart, TL; Garrett, JK; Amer, H; Kennedy, CC	2020	CLINICAL TRANSPLANTATION	34	9	e14017
Can a Left Ventricular Assist Device in Individuals with Advanced Systolic Heart Failure Improve or Reverse Frailty?	Maurer, Mathew S.; Horn, Evelyn; Reyentovich, Alex; Dickson, Victoria Vaughan; Pinney, Sean; Goldwater, Deena; Goldstein, Nathan E.; Jimenez, Omar; Teruya, Sergio; Goldsmith, Jeff; Helmke, Stephen; Yuzefpolskaya, Melana; Reeves, Gordon R.	2017	Journal of the American Geriatrics Society	65	11	2383-2390
Effects of whey protein nutritional supplement on muscle function among community-dwelling frail older people: A multicenter study in China.	Kang, Lin; Gao, Ying; Liu, Xiaohong; Liang, Yinghui; Chen, Yiwen; Liang, Yanhong; Zhang, Lu; Chen, Wei; Pang, Haiyu; Peng, Li-Ning	2019	Archives of Gerontology & Geriatrics	83		7-12

Effects of Protein Supplementation Combined with Exercise Intervention on Frailty Indices, Body Composition, and Physical Function in Frail Older Adults.	Liao, Chun-De; Lee, Pi-Hsia; Hsiao, Dun-Jen; Huang, Shih-Wei; Tsauo, Jau-Yih; Chen, Hung-Chou; Liou, Tsan-Hon	2018	Nutrients	10	12	1916
Effectiveness of exercise interventions on physical function in community-dwelling frail older people: an umbrella review of systematic reviews.	Jadczak, Agathe D.; Makwana, Naresh; Luscombe-Marsh, Natalie; Visvanathan, Renuka; Schultz, Timothy J.	2018	JBI Database of Systematic Reviews & Implementation Reports	16	3	752-775
Multidomain Intervention for Reversal of Cognitive Frailty, Towards a Personalized Approach (AGELESS Trial): Study Design	Ponvel, P; Shahar, S; Singh, DKA; Ludin, AFM; Rajikan, R; Rajab, NF; Ai-Vyrn, C; Din, NC; Ibrahim, N; Subramaniam, P; Haron, H; Ismail, A; Sharif, R; Ramasamy, K; Majeed, AA; Ali, NM; Mohamad, M; Noah, SAM; Ibrahim, AM; Safien, AM; Khalid, NM; Fadzil, NHM; Mangialasche, F; Kivipelto, M	2021	JOURNAL OF ALZHEIMERS DISEASE	82	2	673-687
Functional resistance activities to impact frailty: A protocol for a randomized controlled	Danilovich, MK; Diaz, L; Ciolinio, JD; Corcos, DM	2017	CONTEMPORARY CLINICAL TRIALS COMMUNICATIONS	7		28-32

trial involving home care aide and frail older adult dyads						
Effects of exercise on frailty in patients with hepatocellular carcinoma	Kawaguchi, Takumi; Nakano, Dan; Torimura, Takuji; Koya, Shunji; Hirota, Keisuke; Tsuchihashi, Jin; Koga, Noboru; Narao, Hayato; Tomita, Manabu; Hashida, Ryuki; Matsuse, Hiroo; Sanada, Taku; Notsumata, Kazuo	2020	Hepatology International	14	Supplement 1	S398
"pre-habilitation" of frail candidates for lung transplantation using a mobile health enabled home-based intervention is feasible and safe	Singer, J.P.; Soong, A.; Hays, S.; Kukreja, J.; Bracha, A.; Chin, G.; Wolters, P.J.; Peters, M.; Bruun, A.; Garvey, C.M.	2017	American Journal of Respiratory and Critical Care Medicine	32	6	e13274
Changes in health parameters in older lay volunteers who delivered a lifestyle-based program to frail older people at home	Grabovac, I; Haider, S; Winzer, E; Kapan, A; Schindler, KE; Lackinger, C; Dorner, TE	2018	WIENER KLINISCHE WOCHENSCHRIFT	130	21-22	637-644
Effects of exercise interventions on frailty in pre-maturely aging adults with intellectual disabilities- a preliminary study.	Lin, Shu-Yuan	2021	Disability and health journal		101306633	101105

Community-Based Peer-Led Intervention for Healthy Ageing and Evaluation of the 'HAPPY' Program.	Merchant, Reshma A.; Tsoi, C. T.; Tan, W. M.; Lau, W.; Sandrasageran, S.; Arai, H.	2021	Journal of Nutrition, Health & Aging	25	4	520-527
Getting fit for hip and knee replacement: a protocol for the Fit-Joints pilot randomized controlled trial of a multi-modal intervention in frail patients with osteoarthritis.	Negm, Ahmed M; Kennedy, Courtney C; Ioannidis, George; Gajic-Veljanoski, Olga; Lee, Justin; Thabane, Lehana; Adachi, Jonathan D; Marr, Sharon; Lau, Arthur; Atkinson, Stephanie; Petruccelli, Danielle; DeBeer, Justin; Winemaker, Mitchell; Avram, Victoria; Deheshi, Benjamin; Williams, Dale; Armstrong, David; Lumb, Barry; Panju, Akbar; Richardson, Julie; Papaioannou, Alexandra	2018	Pilot and feasibility studies	4	101676536	127
Effectiveness of Community-Delivered Functional Power Training Program for Frail and Pre-frail Community-Dwelling Older Adults: a Randomized Controlled Study	Tou, NX; Wee, SL; Seah, WT; Ng, DHM; Pang, BWJ; Lau, LK; Ng, TP	2021	PREVENTION SCIENCE	22	8	1048-1059

The effects of a home-based exercise program on physical function in frail older adults.	Matsuda PN; Shumway-Cook A; Ciol MA	2010	Journal of Geriatric Physical Therapy	33	2	78-84
Effects of a 12-Week Exercise Training Program on Physical Function in Institutionalized Frail Elderly.	Ferreira, Cristiane Batisti; Teixeira, PÃ¢mela dos Santos; Alves dos Santos, Geiane; Dantas Maya, Athila Teles; Americano do Brasil, Paula; Souza, VinÃcius Carolino; CÃ³rdova, ClÃ¡udio; Ferreira, Aparecido Pimentel; Lima, Ricardo Moreno; NÃ³brega, OtÃ¡vio de Toledo	2018	Journal of Aging Research			
Treating frailty--a practical guide.	Fairhall, Nicola; Langron, Colleen; Sherrington, Catherine; Lord, Stephen R; Kurrle, Susan E; Lockwood, Keri; Monaghan, Noeline; Aggar, Christina; Gill, Liz; Cameron, Ian D	2011	BMC Medicine	9	1	83-83
New Chronic Obstructive Pulmonary Disease Study Findings Have Been Reported from Qinghai University Affiliated Hospital (Is it possible to reverse frailty in patients	Zhe Wang, Xiaojing Hu, and Qingxiang Dai	2020	Health & Medicine Week	75		e1778

with chronic obstructive pulmonary disease?)						
Exercise as an intervention for frailty.	Liu CK; Fielding RA; Liu, Christine K; Fielding, Roger A	2011	Clinics in Geriatric Medicine	27	1	101-110
Finding new strength. Exercising can help the frail elderly improve quality of life.	Wagner, L	1998	Provider (Washington, D.C.)	24	3	69-70
The effect of self-directed exercise programonphysical function of older adults with frailty	Song, W.; Woo, S.; Oh, S.; Kim, H.; Kim, D.; Kim, J.	2012	Journal of Science and Medicine in Sport	15	SUPPL.1	S80

Wrong Study (n= 51)

Title	Authors	Year	Journal	Volume	Issue	Pages
Effects of physical exercise interventions in frail older adults: a systematic review of randomized controlled trials.	de Labra, Carmen; Guimaraes-Pinheiro, Christyanne; Maseda, Ana; Lorenzo, Trinidad; MillÃ¡n-Calenti, JosÃ© C.	2015	BMC Geriatrics	15		154
Home Exercise Interventions in Frail Older Adults	Stookey, AD; Katzel, LI	2020	CURRENT GERIATRICS REPORTS	9	3	163-175

Interventions Against Disability in Frail Older Adults: Lessons Learned from Clinical Trials.	FougÃ¨re, Bertrand; Morley, J. E.; Little, M. O.; de Souto Barreto, P.; Cesari, M.; Vellas, B.	2018	Journal of Nutrition, Health & Aging	22	6	676-688
Frailty: role of nutrition and exercise.	Kelaiditi, Eirini; van Kan, Gabor Abellan; Cesari, Matteo	2014	Current Opinion in Clinical Nutrition & Metabolic Care	17	1	32-39
Long-term effect of a multicomponent intervention on physical performance and frailty	Lee, H.; Jang, I.; Oh, G.; Jung, H.; Kim, D.	2020	Journal of the American Geriatrics Society	68	SUPPL 1	S140
Falling through the cracks: a case study of how a timely integrated approach can reverse frailty.	Freer, Karen	2020	British Journal of Community Nursing	25	8	382-387
Frailty, Exercise and Nutrition	Michel, JP; Cruz-Jentoft, AJ; Cederholm, T	2015	CLINICS IN LIVER DISEASE	19	3	375-+
Effects of multi-domain interventions in (pre)frail elderly on frailty, functional, and cognitive status: a systematic review	Dedeyne, L; Deschodt, M; Verschueren, S; Tournoy, J; Gielen, E	2017	CLINICAL INTERVENTIONS IN AGING	12		873-896
Physical Exercise for Frailty and Cardiovascular Diseases	Ricci, NA; Cunha, AIL	2020	FRAILTY AND CARDIOVASCULAR DISEASES: RESEARCH INTO AN ELDERLY POPULATION	1216		115-129

Effects of resistance bands exercise for frail older adults: A systematic review and meta-analysis of randomised controlled studies	Saragih, ID; Saragih, IS; Batubara, SO; Yang, YP; Lin, CJ	2022	JOURNAL OF CLINICAL NURSING			43-61
Interventions to prevent or reduce the level of frailty in community-dwelling older adults: a scoping review of the literature and international policies.	PUTS, MARTINE T. E.; TOUBASI, SAMAR; ANDREW, MELISSA K.; ASHE, MAUREEN C.; PLOEG, JENNY; ATKINSON, ESTHER; AYALA, ANA PATRICIA; ROY, ANGELIQUE; MONFORTE, MIRIAM RODRÃ�GUEZ; BERGMAN, HOWARD; MCGILTON, KATHY	2017	Age & Ageing	46	3	383-392
Do home-based exercise interventions improve outcomes for frail older people? Findings from a systematic review.	Clegg, Andrew P; Barber, Sally E; Young, John B; Forster, Anne; Iliffe, Steve J	2012	Reviews in Clinical Gerontology	22	1	68-78
The functional effects of physical exercise training in frail older people: a systematic review.	Chin A Paw MJM; van Uffelen JGL; Riphagen I; van Mechelen W	2008	Sports Medicine	38	9	781-793
The effect of exercise on quality of life and activities of daily life in frail older adults: A systematic review of randomised control trials	Campbell, E; Petermann-Rocha, F; Welsh, P; Celis-Morales, C; Pell, JP; Ho, FK; Gray, SR	2021	EXPERIMENTAL GERONTOLOGY	147		

Exercise prescription to reverse frailty.	Bray, Nick W.; Smart, Rowan R.; Jakobi, Jennifer M.; Jones, Gareth R.	2016	Applied Physiology, Nutrition & Metabolism	41	10	1112-1116
Therapeutic interventions for frail elderly patients: part I. Published randomized trials.	Bibas, Lior; Levi, Michael; Bendayan, Melissa; Mullie, Louis; Forman, Daniel E; Afilalo, Jonathan	2014	Progress in Cardiovascular Diseases	57	2	134-143
Therapeutic Interventions for Frail Elderly Patients: Part II. Ongoing and Unpublished Randomized Trials.	Bendayan, Melissa; Bibas, Lior; Levi, Michael; Mullie, Louie; Forman, Daniel E; Afilalo, Jonathan	2014	Progress in Cardiovascular Diseases	57	2	144-151
Physical therapy treatment on frailty syndrome: systematic review.	Arantes PMM; Alencar MA; Dias RC; Dias JMD; Pereira LSM	2009	Brazilian Journal of Physical Therapy	13	5	365-375
Geroprotectors: A role in the treatment of frailty	Trendelenburg, AU; Scheuren, AC; Potter, P; Muller, R; Bellantuono, I	2019	MECHANISMS OF AGEING AND DEVELOPMENT	180		20-Nov
Outcomes of coordinated and integrated interventions targeting frail elderly people: a systematic review of randomised controlled trials.	Eklund K; Wilhelmson K	2009	Health & Social Care in the Community	17	5	447-458
Reversible Cognitive Frailty, Dementia, and All-Cause Mortality. The	Solfrizzi, Vincenzo; Scafato, Emanuele; Seripa, Davide; Lozupone, Madia; Imbimbo, Bruno P.; D'Amato, Angela;	2017	Journal of the American Medical Directors Association	18	1	89.e1-89.e8

Italian Longitudinal Study on Aging.	Tortelli, Rosanna; Schilardi, Andrea; Galluzzo, Lucia; Gandin, Claudia; Baldereschi, Marzia; Di Carlo, Antonio; Inzitari, Domenico; Daniele, Antonio; SabbÃ , Carlo; Logroscino, Giancarlo; Panza, Francesco					
The role of nutrition and physical activity in frailty: A review	O'Connell, ML; Coppinger, T; McCarthy, AL	2020	CLINICAL NUTRITION ESPEN	35		1-11
A randomized, controlled trial of quadriceps resistance exercise and vitamin D in frail older people: the Frailty Interventions Trial in Elderly Subjects (FITNESS)	Latham NK; Anderson CS; Lee A; Bennett DA; Moseley A; Cameron ID	2003	Journal of the American Geriatrics Society	51	3	291-299
Assessing the feasibility and impact of specially adapted exercise interventions, aimed at improving the multi-dimensional health and functional capacity of frail geriatric hospital inpatients: protocol for a feasibility study	Doody, P; Lord, JM; Greig, CA; Whittaker, AC	2019	BMJ OPEN	9	11	e031159
New Evidence: Mediterranean: Diet Supports Healthy Aging:	Tufts University	2018	Tufts University Health & Nutrition Letter	36	2	

Popular eating pattern may help reduce frailty in older adults.						
The effects of a multi-component intervention on the functional capacity, lower-body muscle strength, balance and gait in frail community-dwelling older people: a randomised controlled trial protocol.	Iosimuta, Natalia C. R.; Pessanha, Fernanda P. A. S; Alves, Natalia; Marques, Larissa T.; Porto, Jaqueline M.; Freire, Renato C.; Ferriolli, Eduardo; de Abreu, Daniela C. C.	2020	European Journal of Physiotherapy	22	5	262-273
Outcomes in a multicomponent exercise programme in frail community-dwelling individuals	Anton, I.; Andueza, E.; Raposo, S.; Embil, X.; Yanguas, J.	2016	European Geriatric Medicine	7	Supplement 1	S115
Introducing an exercise intervention to reverse frailty and build resilience in primary care consultations	Travers, John; Romero-Ortuno, Roman; Cooney, Marie-Therese	2020	European Geriatric Medicine	11	SUPPL 1	S215
Exercise Prescription Intervention Plan for Pre-frail and Frail Elderly in New Taipei City: 1015 Board #276 May 30 3:30 PM - 5:00 PM...American College of Sports Medicine Annual	Lin, Hui-Ping; Tseng, Yu-Ting; Lin, Chi-Hung		Medicine & Science in Sports & Exercise	50		241-241

Meeting, May 29-June 2, 2018, Minneapolis, Minnesota						
Rethink rehabilitation to reverse frailty		2016	Respiratory Therapeutics Week			105
Avoiding frailty as you age: nutritional, physical, and cognitive modifications can help reverse or prevent the condition.(NUTRITION & FITNESS)	Yancy, William S., Jr	2017	Duke Medicine Health News	23	9	6
Can good nutrition, physical training and mental exercises reverse physical frailty in the elderly?	National University of Singapore	2017	Science Daily			
Frailty Intervention Trial (FIT)	Fairhall N; Aggar C; Kurrle SE; Sherrington C; Lord S; Lockwood K; Monaghan N; Cameron ID	2008	BMC Geriatrics	8		27-27
Good nutrition, physical training and mental exercises can reverse physical frailty in the elderly	National University of Singapore	2017	Science Daily			
Protocol for the PREHAB study-Pre-operative Rehabilitation for	Stammers, AN; Kehler, DS; Afilalo, J; Avery, LJ; Bagshaw, SM; Grocott, HP; Legare, JF;	2015	BMJ OPEN	5	3	e007250

reduction of Hospitalization After coronary Bypass and valvular surgery: a randomised controlled trial	Logsetty, S; Metge, C; Nguyen, T; Rockwood, K; Sareen, J; Sawatzky, JA; Tangri, N; Giacomantonio, N; Hassan, A; Duhamel, TA; Arora, RC					
Lifestyle interventions for frail obese older adults	Waters, Debra	2019	Aging Medicine and Healthcare	10	Supplement 1	3
Muscle strength training for reversing frailty: how strong is the evidence?.	Nunan, David	2019	BMJ evidence-based medicine	24	5	199-200
Study protocol of a randomised controlled trial to examine the impact of a complex intervention in pre-frail older adults.	Teh, Ruth; Kerse, Ngaire; Waters, Debra L.; Hale, Leigh; Pillai, Avinesh; Leilua, Evelingi; Tay, Esther; Rolleston, Anna; Edlin, Richard; Maxted, Eruera; Heppenstall, Claire; Connolly, Martin J.	2019	Aging Clinical & Experimental Research	31	10	1407-1417
Biology of frailty: Modulation of ageing genes and its importance to prevent age-associated loss of function	Vina, J; Tarazona-Santabalbina, FJ; Perez-Ros, P; Martinez-Arnau, FM; Borras, C; Olaso-Gonzalez, G; Salvador-Pascual, A; Gomez-Cabrera, MC	2016	MOLECULAR ASPECTS OF MEDICINE	50		88-108
Individualized intervention for frail non-dialysis elderly patients with chronic kidney disease: protocol for a randomized controlled trial.	Chang, Jing; Gao, Yun; Fang, Xiang-Yang; Zhao, Su-Mei; Hou, Yuan-Ping; Sun, Qian-Mei	2020	BMC Geriatrics	20	1	8-Jan

A Multi-Domain Intervention Protocol for the Potential Reversal of Cognitive Frailty: "WE-RISE" Randomized Controlled Trial	Murukesu, RR; Singh, DKA; Shahar, S; Subramaniam, P	2020	FRONTIERS IN PUBLIC HEALTH	8		471
An evaluation of the effectiveness of a multi-modal intervention in frail and pre-frail older people with type 2 diabetes--the MID-Frail study: study protocol for a randomised controlled trial.	RodrÃguez-MaÃ±as, Leocadio; Bayer, Antony J; Kelly, Mark; Zeyfang, Andrej; Izquierdo, Mikel; Laosa, Olga; Hardman, Timothy C; Sinclair, Alan J; Moreira, Severina; Cook, Justin	2014	Trials	15	1	34-34
CONQUERING FRAILTY WITH A MULTI-PERSPECTIVE APPROACH	Aumayr, G	2017	IDIMT-2017 - DIGITALIZATION IN MANAGEMENT, SOCIETY AND ECONOMY	46		211-216
Frailty in olders of a day program outpatients centre	Cruz Santaella, A.; Garcia Castellanos, M.; Torrijos Montalban, A.; Moreno Gonzalez, A.; Cifuentes Caceres, R.; Ortega Herrera, M.; Lozano Serrano, V.	2011	European Geriatric Medicine	2	SUPPL. 1	S113-S114
Diet and Exercise for FRAILty (DEFRAIL): protocol for a study to examine the effect of a novel community-based group exercise and nutritional intervention,	Bambrick, Padraig; Phelan, Niamh; Grant, Emma; Byrne, Thomas; Harrison, Michael; Mulcahy, Riona; Cooke, John	2021	BMJ open	11	6	e042408

designed to reverse frailty in older adults.						
Protocol for a randomised controlled trial of a primary care intervention to Reverse Frailty and Enhance Resilience through Exercise and dietary protein Education (REFEREE) in community-dwelling adults aged 65 and over.	Travers, John; Romero-Ortuno, Roman; Power, Dermot; Doran, Peter; Langan, John; MacNamara, Fergal; McCormack, Darren; McDermott, Christopher; McEntire, Jude; McKiernan, Joanne; Vencken, Sebastian; Murphy, Andrew W; Murphy, Patrick J; Ni She, Eidin; O'Shea, Diarmuid; Cooney, Marie-Therese	2020	HRB open research	3	101754913	91
Efficacy of a primary care geriatric intervention model in reversing frailty-protocol of a randomized clinical trial	Ferreira, Miguel Marques	2018	European Geriatric Medicine	9	Supplement 1	S92
The effect of comprehensive assessment and multi-disciplinary management for the geriatric and frail patient: A multi-center, randomized, parallel controlled trial.	Yao, Simin; Zheng, Peipei; Ji, Liwei; Ma, Zhao; Wang, Lijuan; Qiao, Linlin; Wan, Yuhao; Sun, Ning; Luo, Yao; Yang, Jiefu; Wang, Hua	2020	Medicine	99	46	e22873-e22873
The EX-FRAIL CKD trial: a study protocol for a pilot randomised controlled trial of a home-based	Nixon, AC; Bampouras, TM; Gooch, HJ; Young, HML; Finlayson, KW; Pendleton, N;	2020	BMJ OPEN	10	6	e035344

EXercise programme for pre-frail and FRAIL, older adults with Chronic Kidney Disease	Mitra, S; Brady, ME; Dhaygude, AP					
PREHAB study: a protocol for a prospective randomised clinical trial of exercise therapy for people living with frailty having cancer surgery	McIsaac, DI; Saunders, C; Hladkowicz, E; Bryson, GL; Forster, AJ; Gagne, S; Huang, A; Lalu, M; Lavallee, LT; Moloo, H; Nante, J; Power, B; Scheede-Bergdah, C; Taljaard, M; van Walraven, C; McCartney, CJL	2018	BMJ OPEN	8	6	
Barriers to enrollment in an exercise intervention for pre-frail and frail thoracic surgery patients	Martinchek, M.; Huisingh-Scheetz, M.; Thompson, K.; Pachwicewicz, P.; Ferguson, M.	2016	Journal of the American Geriatrics Society	64	SUPPL. 1	S277

Wrong Type of Intervention/ Frail Population (n= 38)

Title	Authors	Year	Journal	Volume	Issue	Pages
Effects of Home-Based Physical Exercise on Days at Home and Cost-Effectiveness in Pre-Frail and Frail Persons: Randomized Controlled Trial.	Suikkanen, Sara A.; Soukkio, Paula K.; Aartolahti, Eeva M.; Kautiainen, Hannu; KÃ¤Ã¤riÃ¤, Sanna M.; Hupli, Markku T.; SipilÃ¤, Sarianna; PitkÃ¤lÃ¤, Kaisu H.; Kukkonen-Harjula, Katriina T.	2021	Journal of the American Medical Directors Association	22	4	773-779

Preoperative Home-Based Physical Therapy Versus Usual Care to Improve Functional Health of Frail Older Adults Scheduled for Elective Total Hip Arthroplasty: A Pilot Randomized Controlled Trial.	Oosting, Ellen; Jans, Marielle P.; Dronkers, Jaap J.; Naber, Roelfrieke H.; Dronkers-Landman, Christa M.; Appelman-de Vries, Suzan M.; van Meeteren, Nico L.	2012	Archives of Physical Medicine & Rehabilitation	93	4	610-616
Home-based preoperative rehabilitation (prehab) to improve physical function and reduce hospital length of stay for frail patients undergoing coronary artery bypass graft and valve surgery.	Waite, Iain; Deshpande, Ranjit; Baghai, Max; Massey, Tania; Wendler, Olaf; Greenwood, Sharlene	2017	Journal of Cardiothoracic Surgery	12		1-7
The Characteristic of a Simple Exercise Program under the Instruction of Physiotherapists-For General Elderly People and Frail Elderly People	Nakagawa, K; Inomata, N; Konno, Y; Nakazawa, R; Hagiwara, K; Sakamoto, M	2008	JOURNAL OF PHYSICAL THERAPY SCIENCE	20	4	197-203
The effects of exercise and protein-energy supplements on body composition and muscle function in frail elderly individuals: a long-term controlled randomised study	Bonnefoy, M; Cornu, C; Normand, S; Boutitie, F; Bugnard, F; Rahmani, A; Lacour, JR; Laville, M	2003	BRITISH JOURNAL OF NUTRITION	89	5	731-738

Feasibility and effectiveness of a cosmetic intervention program for institutionalized older women in Japan.	Hayakawa, Yohko; Shoji, Ikuko; Kumon, Hiroko; Tokita, Masumi; Kamata, Masazumi; Arao, Takashi	2016	Preventive medicine reports	4	101643766	242-7
A home-based exercise program focused on proprioception to reduce falls in frail and pre-frail community-dwelling older adults.	PÃ©rez-Ros, Pilar; Vila-Candel, Rafael; MartÃnez-Arnau, Francisco Miguel	2020	Geriatric Nursing	41	4	436-444
Older persons with signs of frailty in a home-based physical exercise intervention: baseline characteristics of an RCT.	Suikkanen, Sara; Soukkio, Paula; PitkÃ¤lÃ¤, Kaisu; KÃ¤Ã¤riÃ¤, Sanna; Kautiainen, Hannu; SipilÃ¤, Sarianna; Kukkonen-Harjula, Katriina; Hupli, Markku	2019	Aging Clinical & Experimental Research	31	10	1419-1427
Effect of a multifactorial interdisciplinary intervention on mobility-related disability in frail older people: randomised controlled trial.	Fairhall, Nicola; Sherrington, Catherine; Kurrle, Susan E; Lord, Stephen R; Lockwood, Keri; Cameron, Ian D	2012	BMC Medicine	10	1	120-120
Effects of Whole-Body Vibration Training on the Physical Function of the Frail Elderly: An Open, Randomized Controlled Trial.	Wadsworth, Daniel; Lark, Sally	2020	Archives of Physical Medicine & Rehabilitation	101	7	1111-1119

Effects of exercise training added to ongoing hormone replacement therapy on bone mineral density in frail elderly women.	Villareal DT; Binder EF; Yarasheski KE; Williams DB; Brown M; Sinacore DR; Kohrt WM	2003	Journal of the American Geriatrics Society	51	7	985-990
The effects of a life goal-setting technique in a preventive care program for frail community-dwelling older people: a cluster nonrandomized controlled trial.	Yoshimi Yuri; Shinichi Takabatake; Tomoko Nishikawa; Mari Oka; Taro Fujiwara; Yuri, Yoshimi; Takabatake, Shinichi; Nishikawa, Tomoko; Oka, Mari; Fujiwara, Taro	2016	BMC Geriatrics	16		1-11
A pilot randomized controlled trial to improve geriatric frailty.	Chan, Ding-Cheng Derrick; Tsou, Hsiao-Hui; Yang, Rong-Sen; Tsauo, Jau-Yih; Chen, Ching-Yu; Hsiung, Chao Agnes; Kuo, Ken N	2012	BMC Geriatrics	12	1	58-58
Additive Role of a Potentially Reversible Cognitive Frailty Model and Inflammatory State on the Risk of Disability: The Italian Longitudinal Study on Aging	Italian Longitudinal Study Aging; Solfrizzi, V; Scafato, E; Lozupone, M; Seripa, D; Giannini, M; Sardone, R; Bonfiglio, C; Abbrescia, DI; Galluzzo, L; Gandin, C; Baldereschi, M; Di Carlo, A; Inzitari, D; Daniele, A; Sabba, C; Logroscino, G; Panza, F	2017	AMERICAN JOURNAL OF GERIATRIC PSYCHIATRY	25	11	1236-1248
Progressive functional wii-hab in pre-frail older adults	Daniel, K.M.; Cason, C.; Ray, C.	2011	Journal of the American Geriatrics Society	59	SUPPL. 1	S157

Nutritional interventions to prevent and treat frailty.	Cruz-Jentoft, Alfonso J.; Woo, Jean	2019	Current Opinion in Clinical Nutrition & Metabolic Care	22	3	191-195
Effectiveness of a community-wide intervention for population-level frailty and functional health in older adults: A 2-year cluster nonrandomized controlled trial.	Seino, Satoshi; Tomine, Yui; Nishi, Mariko; Hata, Toshiki; Fujiwara, Yoshinori; Shinkai, Shoji; Kitamura, Akihiko	2021	Preventive Medicine	149		N.PAG-N.PAG
Effectiveness of interventions to prevent pre-frailty and frailty progression in older adults: a systematic review.	ApÃ³stolo, JoÃ£o; Cooke, Richard; Bobrowicz-Campos, Elzbieta; Santana, Silvina; Marcucci, Maura; Cano, Antonio; Vollenbroek-Hutten, Miriam; Germini, Federico; D'Avanzo, Barbara; Gwyther, Holly; Holland, Carol	2018	JBI Database of Systematic Reviews & Implementation Reports	16	1	140-232
Effectiveness of an individually tailored home-based exercise rogramme for pre-frail older adults, driven by a tablet application and mobility monitoring: a pilot study	Geraedts, HAE; Dijkstra, H; Zhang, W; Ibarra, F; Far, IK; Zijlstra, W; Stevens, M	2021	EUROPEAN REVIEW OF AGING AND PHYSICAL ACTIVITY	18	1	
Effectiveness of an intervention to prevent frailty in pre-frail community-dwelling older	SERRA-PRAT, M.; SIST, X.; DOMENICH, R.; JURADO, L.; SAIZ, A.; ROCES, A.; PALOMERA,	2017	Age & Ageing	46	3	401-407

people consulting in primary care: a randomised controlled trial.	E.; TARRADELLES, M.; PAPIOL, M.					
Effects of elastic band exercise on the frailty states in pre-frail elderly people.	Chen, Rujie; Wu, Qingwen; Wang, Dongyan; Li, Zhou; Liu, Howe; Liu, Guangtian; Cui, Ying; Song, Linlin	2020	Physiotherapy Theory & Practice	36	9	1000-1008
Effectiveness of an intervention to prevent frailty in pre-frail community-dwelling older people consulting in primary care: a randomised controlled trial	Serra-Prat, M; Sist, X; Domenich, R; Jurado, L; Saiz, A; Roces, A; Palomera, E; Tarradelles, M; Papiol, M	2017	AGE AND AGEING	46	3	401-407
MULTI-COMPONENT EXERCISE WITH HIGH-INTENSITY, FREE-WEIGHT, FUNCTIONAL RESISTANCE TRAINING IN PRE-FRAIL FEMALES: A QUASI-EXPERIMENTAL, PILOT STUDY	Bray, NW; Jones, GJ; Rush, KL; Jones, CA; Jakobi, JM	2020	JOURNAL OF FRAILTY & AGING	9	2	111-117
Association between frailty and delirium in older adult patients discharged from hospital	Verloo, H; Goulet, C; Morin, D; von Gunten, A	2016	CLINICAL INTERVENTIONS IN AGING	11		55-63

Rodent models of frailty and their application in preclinical research	Banga, S; Heinze-Milne, SD; Howlett, SE	2019	MECHANISMS OF AGEING AND DEVELOPMENT	179		1-10
Frailty and Sarcopenia in Older Patients Receiving Kidney Transplantation	Gandolfini, I; Regolisti, G; Bazzocchi, A; Maggiore, U; Palmisano, A; Piotti, G; Fiaccadori, E; Sabatino, A	2019	FRONTIERS IN NUTRITION	6		169
"I Don't Feel Like Myself".	Firnhaber, Gina C.; Kolasa, Kathryn M.	2016	Nutrition Today	51	6	281-289
Allicin Reversed the Process of Frailty in Aging Male Fischer 344 Rats With Osteoporosis.	Liu, Yang; You, Meigui; Shen, Jianwei; Xu, Yaping; Li, Lin; Wang, Dongtao; Yang, Yajun	2020	Journals of Gerontology Series A: Biological Sciences & Medical Sciences	75	5	821-825
Voluntary Aerobic Exercise Reverses Frailty in Old Mice.	Graber, Ted G; Ferguson-Stegall, Lisa; Liu, Haiming; Thompson, LaDora V	2015	Journals of Gerontology Series A: Biological Sciences & Medical Sciences	70	9	1045-1058
Effectiveness of an intervention in multicomponent exercise in primary care to improve frailty parameters in patients over 70â€‰years of age (MEFAP-project), a randomised clinical trial: rationale and study design.	Castell, M. V.; GutiÃ©rrez-Misis, A.; SÃ¡nchez-MartÃnez, M.; Prieto, M. A.; Moreno, B.; NuÃ±ez, S.; Triano, R.; de Antonio, M. P.; Mateo, C.; Cano, M. D.; Garrido, A.; Julian, R.; Polentinos, E.; Rodriguez-Barrientos, R.; Otero Puime, A.	2019	BMC Geriatrics	19	1	9-Jan
Frail older adultsâ€™ perceptions of an in-	Oâ€™Hare, Louise; Savage, Eileen; McCullagh, Ruth; Bantry	2017	Physiotherapy	103	4	478-484

hospital structured exercise intervention.	White, Eleanor; Fitzgerald, Eilis; Timmons, Suzanne					
Effectiveness of the PROMUFRA program in pre-frail, community-dwelling older people: A randomized controlled trial.	Barrachina-Igual, JoaquÃn; MartÃnez-Arnau, Francisco M.; PÃ©rez-Ros, Pilar; Flor-Rufino, Cristina; Sanz-Requena, Roberto; Pablos, Ana	2021	Geriatric Nursing	42	2	582-591
Frail older adults' perceptions of an in-hospital structured exercise intervention	Broderick, L.; Savage, E.; McCullagh, R.; Bantry-White, E.; Timmons, S.	2013	Irish Journal of Medical Science	182	SUPPL. 6	S240
Implementation and Evaluation of a Fall Risk Screening Strategy Among Frail Older Adults for the Primary Care Setting: A Study Protocol	Meekes, WMA; Leemrijse, CJ; Korevaar, JC; Henquet, JMAE; Nieuwenhuis, M; van de Goor, LAM	2020	CLINICAL INTERVENTIONS IN AGING	15		1625-1636
Corrigendum: Effectiveness of interventions to prevent pre-frailty and frailty progression in older adults: A systematic review (JBI Database of Systematic Reviews and Implementation Reports (2018) 16:1 (140-232) DOI: 10.11124/JBISRIR-2017-003382)	Apostolo, Joao; Bobrowicz-Campos, Elzbieta; Cooke, Richard; Gwyther, Holly; Holland, Carol; Santana, Silvina; Marcucci, Maura; Germini, Federico; Cano, Antonio; Vollenbroek-Hutten, Miriam; D'Avanzo, Barbara	2018	JBI Database of Systematic Reviews and Implementation Reports	16	5	1282-1283

Functional and cognitive impairment prevention through early physical activity for geriatric hospitalized patients: study protocol for a randomized controlled trial.	MartÃnez-Velilla, NicolÃ¡s; Casas-Herrero, Alvaro; Zambom-Ferraresi, FabrÃcio; SuÃ¡rez, Nacho; Alonso-Renedo, Javier; ContÃn, Koldo Cambra; de Asteasu, Mikel LÃ³pez-SÃ¡ez; Echeverria, Nuria Fernandez; LÃ¡zaro, MarÃa Gonzalo; Izquierdo, Mikel	2015	BMC Geriatrics	15	1	9-Jan
Extracellular vesicles from young mice improve physiological functions and reverse frailty in aged mice	Borras, C.	2021	European Journal of Clinical Investigation	51	SUPPL 1	123
Approach to frailty in the elderly in primary care and the community.	Christine Yuanxin Chen; Peiying Gan; Choon How How; Chen, Christine Yuanxin; Gan, Peiying; How, Choon How	2018	Singapore Medical Journal	59	5	240-245
