# Supplementary material for: Reversing frailty in older adults: a scoping review
Source: BMC Geriatr. 2023 Nov 17;23:751. doi: 10.1186/s12877-023-04309-y (PMC10655301; doi:10.1186/s12877-023-04309-y)
Supplement: Supplementary file 3 — Supplementary Material 3 [file 12877_2023_4309_MOESM3_ESM.pdf]

Data Extraction Items

Extraction categories	Data items
Bibliography	Authors, journal title, year of publication, corresponding author contact.
Study characteristics	Aim of study, research design, country of study, setting of the study, conceptual framework of frailty considered, domains of frailty considered.
Participant information	Age of participants, Specific health conditions, the total number enrolled in the study, the total number lost to follow-up, frail state of participants at the start of the study, sex of participants.
Intervention characteristics	Reversing/treating frailty mentioned, theory guiding intervention, types of Interventions, who performed an intervention, how was intervention provided, frequency of intervention, duration of follow-up post-intervention (months), outcome and outcome measures.
Quality Appraisal	JBI quality appraisal tool, study limitations.
