# Supplementary material for: Reversing frailty in older adults: a scoping review
Source: BMC Geriatr. 2023 Nov 17;23:751. doi: 10.1186/s12877-023-04309-y (PMC10655301; doi:10.1186/s12877-023-04309-y)
Supplement: Supplementary file 4 — Supplementary Material 4 [file 12877_2023_4309_MOESM4_ESM.pdf]

Interpretation of quality assessment scores

Study Design	Total score	Interpretation
Randomized Control Trial	13	10-13: Low risk of bias<br>6-9: Moderate risk of bias<br>0-5: High risk of bias
Quasi-Experimental study	9	8-9: Low risk of bias<br>4-7: Moderate risk of bias<br>0-3: High risk of bias
Cohort Study	11	9-11: Low risk of bias<br>5-8: Moderate risk of bias<br>0-4: High risk of bias
Case Series	10	8-10: Low risk of bias<br>5-7: Moderate risk of bias<br>0-4: High risk of bias
Case report	8	7-8: Low risk of bias<br>4-6: Moderate risk of bias<br>0-3: High risk of bias

Quality Assessment scores of individual studies

Author	Year	Research design	Score	Interpretation
Arrieta	2019	RCT	12	Low risk of bias
Brown	2000	RCT	8	Moderate risk of bias
Cameron	2013	RCT	7	Moderate risk of bias
Cesari	2015	RCT	8	Moderate risk of bias
Chin A Paw	2001	RCT	9	Moderate risk of bias
Coelho-Junior	2021	RCT	9	Moderate risk of bias
de Souto Barreto	2018	RCT	10	Low risk of bias
Fiatarone	1994	RCT	8	Moderate risk of bias
Imaoka	2016	RCT	8	Moderate risk of bias
Kim	2015	RCT	9	Moderate risk of bias
Lammes	2012	RCT	7	Moderate risk of bias
Li	2010	RCT	10	Low risk of bias
Liao	2019	RCT	12	Low risk of bias
Nagai	2018	RCT	9	Moderate risk of bias
Ng	2017	RCT	10	Low risk of bias
Ng	2015	RCT	10	Low risk of bias
Rydwik	2010	RCT	9	Moderate risk of bias
Sadjapong	2020	RCT	12	Low risk of bias
Sahin	2018	RCT	9	Moderate risk of bias
Seino	2017	RCT	10	Low risk of bias
Tarazona-Santabalbina	2016	RCT	12	Low risk of bias
Torres-Sánchez	2017	RCT	10	Low risk of bias
Vestergaard	2008	RCT	8	Moderate risk of bias
Cadore	2014	Case Series	9	Low risk of bias
Kim	2020	Case Series	8	Low risk of bias
Hergott	2020	Case Report	6	Moderate risk of bias
Larsen	2020	Cohort Studies	9	Low risk of bias
Takatori	2021	Cohort Studies	9	Low risk of bias
Abizanda	2015	Cohort Studies	7	Moderate risk of bias
Liu	2017	Quasi-Experimental Studies	8	Low risk of bias
Losa-Reyna	2019	Quasi-Experimental Studies	8	Low risk of bias
Oh	2021	Quasi-Experimental Studies	9	Low risk of bias
Ushijima	2021	Quasi-Experimental Studies	9	Low risk of bias
